# Pancreatic islet **α** cell function and proliferation require the arginine transporter SLC7A2

**DOI:** 10.1172/JCI173913

**Published:** 2026-06-15

**Authors:** Erick Spears, Jade E. Stanley, Matthew Shou, Linlin Yin, Xuan Li, Chunhua Dai, Amber Bradley, Katelyn Sellick, Greg Poffenberger, Katie C. Coate, Shristi Shrestha, Anna Marie R. Schornack, Taverlyn Shepard, Madushika Wimalarathne, Regina Jenkins, Kyle W. Sloop, Keith T. Wilson, Alan D. Attie, Mark P. Keller, Wenbiao Chen, Alvin C. Powers, E. Danielle Dean

**Affiliations:** 1 Division of Diabetes, Endocrinology, and Metabolism, Department of Medicine, Vanderbilt University Medical Center, Nashville, Tennessee, USA.; 2 Department of Biology, Belmont University, Nashville, Tennessee, USA.; 3 Department of Molecular Physiology & Biophysics, Vanderbilt University, Nashville, Tennessee, USA.; 4 Department of Biochemistry, University of Wisconsin–Madison, Madison, Wisconsin, USA.; 5 Diabetes and Complications, Lilly Research Laboratories, Eli Lilly and Company, Indianapolis, Indiana, USA.; 6 Division of Gastroenterology, Hepatology & Nutrition, Department of Medicine;; 7 Department of Pathology, Microbiology & Immunology;; 8 Center for Mucosal Inflammation & Cancer; and; 9 Program in Cancer Biology, Vanderbilt University School of Medicine, Nashville, Tennessee, USA.; 10 Veterans Affairs Tennessee Valley Healthcare System, Nashville, Tennessee, USA.

**Keywords:** Cell biology, Endocrinology, Metabolism, Amino acid metabolism, Insulin, Islet cells

## Abstract

Interrupting glucagon signaling decreases gluconeogenesis and the fractional extraction of amino acids by liver from blood, resulting in lower glycemia. The resulting hyperaminoacidemia stimulates α cell proliferation and glucagon secretion via a liver/α cell axis. We hypothesized that α cells detect and respond to circulating amino acids’ levels via a unique amino acid transporter repertoire. We found that *Slc7a2/SLC7A2* is the most highly expressed cationic amino acid transporter in α cells, with its expression being 3-fold greater in α than β cells in both mouse and human. Employing cell culture, zebrafish, and knockout mouse models, we found that the cationic amino acid arginine and SLC7A2 are required for α cell proliferation in response to interrupted glucagon signaling. Ex vivo and in vivo assessment of islet function in *Slc7a2^–/–^* mice showed decreased arginine-stimulated glucagon and insulin secretion. We found that arginine activation of mTOR signaling and induction of the glutamine transporter SLC38A5 was dependent on SLC7A2, showing that the role of both in α cell proliferation is dependent on arginine transport and SLC7A2. Finally, we identified single nucleotide polymorphisms in *SLC7A2* associated with HbA1c. Together, these data indicate a central role for SLC7A2 in amino acid–stimulated α cell proliferation and islet hormone secretion.

## Abstract 2

Spears et al. show that the arginine transporter SLC7A2 plays a critical role in the regulation of insulin and glucagon secretion and alpha cell proliferation.

## Introduction

Pancreatic α cells secrete glucagon, which stimulates gluconeogenesis and glycogenolysis in the liver when blood glucose concentrations are low. When glucose levels rise, β cells secrete insulin, which counteracts these effects. The counterregulatory relationship between these 2 hormones is essential to maintenance of glucose homeostasis. The development of type 2 diabetes (T2D) results from reduced insulin secretion from pancreatic islets and diminished insulin action in peripheral tissues. However, hyperglucagonemia in T2D, due to the inability to suppress glucagon secretion, also promotes T2D-associated hyperglycemia, highlighting a central role for α cell dysregulation in addition to insulin insufficiency (1). Interrupting glucagon action lowers blood glucose under non-diabetic conditions and in rodent models and humans with diabetes (2–5). Therefore, glucagon receptor antagonism is under investigation as a therapeutic approach to treat both type 1 diabetes (T1D) and T2D (6–8). Studies of glucagon antagonism led to the discovery of an interaction between the α cells in the pancreatic islet and hepatocytes that has been termed the liver/α cell axis. Interrupted glucagon signaling decreases hepatic amino acid extraction from the circulation and catabolism, resulting in hyperaminoacidemia. This in turn feeds back to α cells, stimulating them to proliferate and secrete more glucagon. This response to interrupted glucagon signaling by any approach (e.g., small molecule antagonist, genetic deletion of the glucagon gene, glucagon receptor, or downstream signaling components) is conserved in zebrafish, mouse, and human islets (9–14).

Most recently, studies have identified high glutamine as being important for this amino acid–stimulated α cell proliferation and involving the mTOR-dependent upregulation of expression of the glutamine transporter *Slc38a5* (9, 10). These studies also indicated roles for other amino acids in the liver/α cell axis, as elevated levels of glutamine were essential, but not sufficient, suggesting a role for a combination of other amino acids, like those found in serum of mice with interrupted glucagon signaling (10). Furthermore, global loss of *Slc38a5* expression reduced, but did not completely prevent, α cell proliferation following glucagon signaling interruption (9).

Arginine has profound physiological effects on islet cells as a potent secretagogue for both insulin and glucagon (15–19). The most common arginine transporters belong to the Slc7a subfamily of y^+^-type cationic amino acid transporters historically known as CAT proteins (SLC7A1–4 and SLC7A14) (20). Of all these subfamily members, *SLC7A2* is the most highly expressed in human pancreas and liver (https://gtexportal.org/home/). In mice, *Slc7a2* expression was decreased in the liver in response to interrupted glucagon signaling, and this was accompanied by an increase in serum arginine (9, 10). Though SLC7A2 and arginine transport have been studied in other tissues and cell types, including macrophages, astrocytes, lung, and intestine (21–24), its role in islet cell physiology has not been assessed.

These observations led us to hypothesize that arginine plays a central role in the liver/α cell axis and that SLC7A2 is the primary arginine transporter in α cells. Here, we show that *Slc7a2* is highly expressed in α and β cells from humans, mice, and zebrafish and that arginine is required for amino acid–stimulated α cell proliferation. Using genetic loss-of-function models in zebrafish and mice, we demonstrate that SLC7A2 is required for amino acid–stimulated α cell proliferation following interrupted glucagon signaling and that it plays a critical role in arginine-stimulated insulin and glucagon secretion from pancreatic β or α cells, respectively. Finally, we demonstrated that *Slc7a2* expression is required for the upregulation of *Slc38a5* expression following interrupted glucagon signaling. Together, these studies reveal a conserved role for the arginine transporter SLC7A2 in amino acid–regulated islet cell biology.

## Results

### Arginine is necessary for amino acid–stimulated α cell proliferation in vitro.

**Figure 1:**
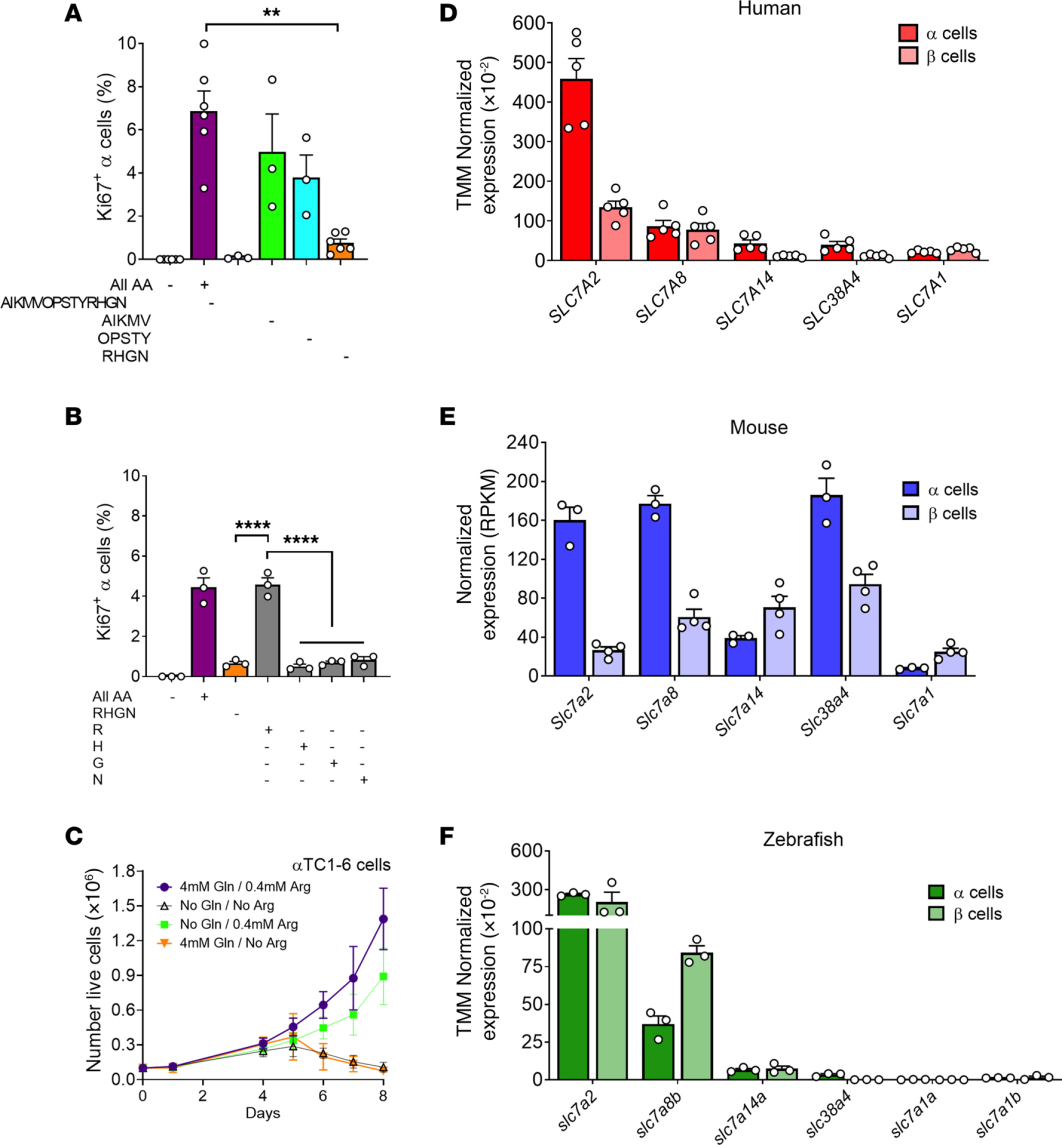
Arginine stimulates α cell proliferation in cultured mouse islets and cationic amino acid transporter, *Slc7a2*, expression is conserved in human, mouse, and zebrafish α cells. (**A**) Ex vivo α cell proliferation analysis in low (All AA-) and high (All AA+) amino acid–containing medium; medium with low concentrations of amino acids (AIKMVOPSTYRHGN-); high AA medium with alanine (A), isoleucine (I), lysine (K), methionine (M), and valine (V) at low concentrations (AIKMV-); high AA medium with ornithine (O), proline (P), serine (S), threonine (T), and tryptophan (Y) at low concentrations (OPSTY-); AA medium with arginine (R), histidine (H), glycine (G), and asparagine (N) at low concentrations (RHGN–). (**B**) Ex vivo α cell proliferation analysis in low (–) and high (+) amino acid–containing medium, in otherwise high AA medium with low RHGN concentrations (orange bar, RHGN-), and with each of the low-concentration AAs in low RHGN medium restored to high levels (gray bars, R+, H+, G+, and N+). We determined α cell proliferation by percent Ki67^+^/Gcg^+^ cells per total Gcg^+^ cells in isolated islets cultured in medium containing different variations of amino acid concentrations (*n* = 3 per group). AA = all amino acids, R = arginine, H = histidine, G = glycine, N = asparagine, + = high AA concentrations (equivalent to that in serum of *Gcgr*^–/–^ mice), – = low AA concentration (equivalent to that in serum of *Gcgr^+/+^*). (**C**) Cell growth over time of αTC1-6 cultured cells in control DMEM or in DMEM lacking glutamine (Gln), arginine (Arg), or both. (**D**–**F**) Comparison of cationic amino acid transporter expression in α and β cells from published (**D**) human (*n* = 5) (25, 26), (**E**) mouse (*n* = 3–4) (27), and (**F**) zebrafish (*n* = 3) (28) RNA-seq datasets. Expression of other cationic amino acid transporter not shown was below the limits of detection in islet cells. Significance was designated: **P < 0.005, and ****P < 0.0001.

Previous studies demonstrated the importance of hyperaminoacidemia for α cell proliferation and specifically a role for glutamine and its major transporter, SLC38A5, in mice and zebrafish (9, 10). Since *Slc38a5* expression differs between mouse and human α cells and its ablation only partially suppressed α cell proliferation (9, 10), we evaluated whether other amino acids are individually required for amino acid–stimulated α cell proliferation, as is glutamine. As described previously, culturing isolated mouse islets in high amino acid medium increased α cell proliferation (Figure 1A) (10). This high amino acid medium (All AA +) mimicked levels in mice with interrupted glucagon signaling while low amino acid medium (All AA –) was similar to levels in wild-type mouse serum (Supplemental Table 1; supplemental material available online with this article; https://doi.org/10.1172/JCI173913DS1). To identify other critical amino acids, we adopted a 2-step approach to minimize the number of islets in the screen. As a first step, we reduced the levels of groups of 4 amino acids in the high amino acid medium (Supplemental Table 1). Only islets cultured in medium with low arginine, histidine, glycine, and asparagine (RHGN^lo^) had decreased α cell proliferation (Figure 1A). To determine if a low level of a single amino acid in the RHGN^lo^ medium was responsible, we then individually replaced each of these 4 amino acids in the RHGN^lo^ medium at the high concentration and found that only arginine (R) stimulated amino acid–stimulated α cell proliferation (Figure 1B). We also assessed the importance of arginine for proliferation of a cultured α cell line, αTC1-6 cells. Culturing these cells in their normal DMEM-based medium lacking arginine inhibited proliferation (Figure 1C and Supplemental Table 2). These results indicate that arginine, in addition to glutamine, is required for α cell proliferation (10).

### Slc7a2 is highly expressed in pancreatic α and β cells.

Because interruption of glucagon signaling leads to increased serum amino acids and increased α cell proliferation (9, 10), we evaluated the expression of cationic amino acid transporters in pancreatic α and β cells by mining published transcriptomics datasets. In human RNA-seq datasets (25, 26), *SLC7A2* is the most highly expressed of all SLC (SoLute Carrier) superfamily genes in α cells, and its expression is 3-fold higher in α cells than in β cells (Figure 1D and Supplemental Table 3). From mouse RNA-seq data (27), *Slc7a2* is one of the most highly expressed amino acid transporters, with a 6-fold greater expression in α cells when compared with β cells (Figure 1E and Supplemental Table 3). In zebrafish (28), *slc7a2* is the third most highly expressed amino acid transporter (Supplemental Table 3) but is the most highly expressed cationic amino acid transporter (Figure 1F). The greater level of *Slc7a2* expression in islet cells of humans, mice, and zebrafish points toward its evolutionarily conserved importance in the endocrine pancreas and α cells.

### SLC7A2 is associated with hemoglobin A1c levels in humans.

**Figure 2:**
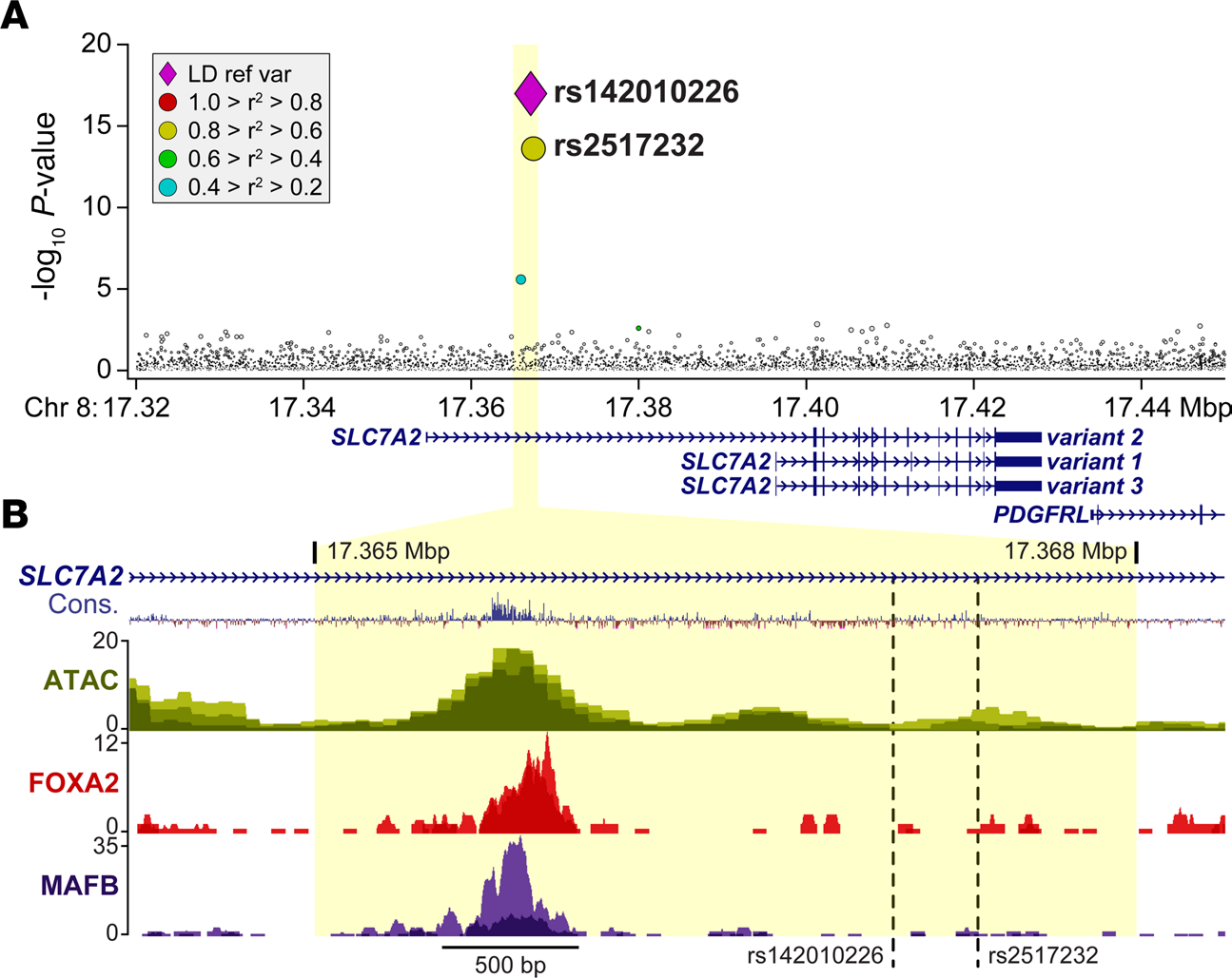
GWAS analysis of the SLC7A2 gene locus identifies SNPs associated with HbA1c in human. (**A**) SNPs significantly associated with the human SLC7A2 gene locus with HbA1c levels are represented by a violet diamond (rs142010226) and a yellow (rs2517232) circle. Gray circles represent SNPs not significantly associated with HbA1c or for which there are no associated data, respectively. Yellow shading indicates the region identified in **B**. LD, linkage disequilibrium. (**B**) ATAC-seq analyses, from Pasquali et al. (29), of the SLC7A2 locus in human islets with highly conserved regions (blue, Cons.), ATAC peaks in green, FOXA2 binding in red, and MAFB binding in purple.

To ask if *SLC7A2* contributes to nutrient homeostasis in humans, we investigated if the *SLC7A2* gene locus is associated with diabetes-related phenotypes in human GWAS. Two single nucleotide polymorphisms (SNPs) have been identified within the first intron of *SLC7A2* that are associated with hemoglobin A1c (HbA1c) levels (Figure 2A). rs142010226 (chr 8, 17367112:A/G) and rs2517232 (chr 8, 17367421:A/G) are both strongly associated (*P* < 10^–15^) with HbA1c in the EXTEND human cohort, which consists of 1,395 diabetic and 5,764 non-diabetic individuals of European ancestry, which we identified via data mining the T2D Knowledge Portal (29). Further data mining of published ATAC-seq and ChIP-seq datasets from human islets (Figure 2B) showed that both SNPs occur within ~1 kb of binding sites for MAFB and FOXA2, 2 transcription factors (30, 31) that have previously been shown to be critical for α cell gene expression (32–35). In summary, these findings are consistent with a role for *SLC7A2* in glucose homeostasis in humans and suggest that genetic variants associated with HbA1c may influence MAFB- and/or FOXA2-dependent regulation of *SLC7A2* expression in islets.

### Slc7a2^–/–^ mice have decreased arginine-stimulated glucagon and insulin secretion.

**Figure 3:**
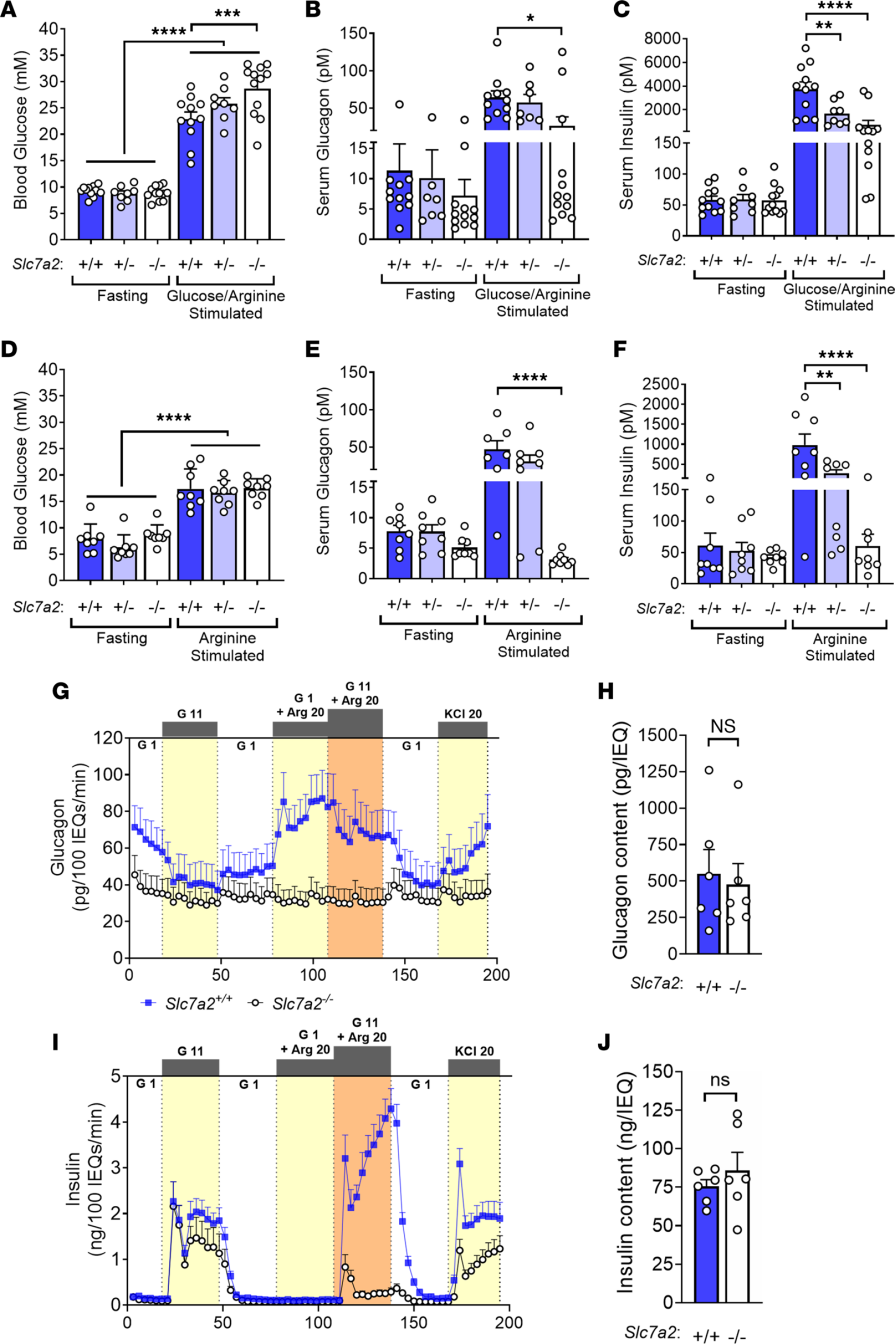
*Slc7a2^–/–^* mice have decreased glucagon and insulin secretion under highly stimulatory conditions. (**A**) Blood glucose, (**B**) serum glucagon, and (**C**) serum insulin for *Slc7a2^+/+^* (*n* = 11), *Slc7a2^+/–^* (*n* = 8), and *Slc7a2^–/–^* (*n* = 12) mice fasted for 6 hours, injected with glucose/arginine bolus, and sampled 15 minutes after injection. (**D**) Blood glucose, (**E**) serum glucagon, and (**F**) serum insulin for *Slc7a2^+/+^*, *Slc7a2^+/–^*, and *Slc7a2^–/–^* mice fasted for 6 hours, injected with arginine bolus, and sampled 15 minutes after injection (*n* = 8 each genotype). (**G**) Glucagon secretion, (**H**) glucagon content, (**I**) insulin secretion, and (**J**) insulin content as assessed by perifusion of islets isolated from *Slc7a2^+/+^* and *Slc7a2^–/–^* mice (*n* = 4 each genotype). G 1 = 1 mM glucose, G 11 = 11 mM glucose, Arg 20 = 20 mM arginine, KCl 20 = 20 mM KCl. *P < 0.05, **P < 0.005, ***P < 0.0005, and ****P < 0.0001.

To understand the role of arginine transport in islet function and α cell proliferation, we examined the impact of loss of *Slc7a2* on glucose homeostasis and islet function using a global *Slc7a2*-knockout (*Slc7a2^–/–^*)mouse line (21–23, 36). We found that *Slc7a2^–/–^* mice had normal glucose tolerance in response to intraperitoneal glucose injection (Supplemental Figure 1, A and B). To assess stimulated islet hormone secretion and glycemic regulation, we gave *Slc7a2^–/–^* mice an intraperitoneal glucose/arginine bolus, and blood glucose, serum glucagon, and serum insulin concentrations were measured (Figure 3, A–C). Stimulated blood glucose levels were higher in *Slc7a2^–/–^* than in *Slc7a2^+/+^* mice (Figure 3A), supporting a role for SLC7A2 in glycemic regulation. Serum glucagon levels increased similarly in *Slc7a2^+/+^* and *Slc7a2^+/–^* after stimulation with glucose and arginine. However, stimulated glucagon levels in *Slc7a2^–/–^* mice were 60% lower than their *Slc7a2^+/+^* and *Slc7a2^+/–^* littermates and not different from fasted glucagon levels in the same *Slc7a2^–/–^* mice (Figure 3B). Similar to glucose/arginine bolus, intraperitoneal administration of arginine alone did not increase serum glucagon in Slc7a2^–/–^ animals, as stimulated values were 95% lower than *Slc7a2^+/+^* mice and not significantly different from fasted glucagon values in the same mice (Figure 3E), indicating a central role for SLC7A2 in arginine-stimulated glucagon secretion.

Interestingly, glucose/arginine-stimulated serum insulin levels were similarly decreased in *Slc7a2^–/–^* animals, but there was also a decrease in glucose/arginine-stimulated serum insulin in *Slc7a2^+/–^* animals (Figure 3C). Intraperitoneal administration of arginine resulted in no change in blood glucose for *Slc7a2^–/–^* compared with *Slc7a2^+/+^* and *Slc7a2^+/–^* mice (Figure 3D). Arginine did not increase serum insulin levels in *Slc7a2^–/–^* mice, and arginine-stimulated insulin levels were also lower in *Slc7a2^+/–^* mice (Figure 3F). These data indicate that arginine transport via SLC7A2 is necessary for arginine-stimulated glucagon and insulin secretion. Of note, the decrease in arginine-stimulated insulin secretion in Slc7a2^+/–^ mice indicates that arginine transport may be the rate limiting step in stimulated glucagon and insulin secretion.

### Slc7a2^–/–^ mouse islets have an islet-intrinsic impaired response to arginine resulting in defective glucagon secretion.

Because we used a global *Slc7a2*-knockout mouse, we assessed whether the observed defects in arginine-stimulated secretion in vivo were due to intrinsic islet SLC7A2 loss (islet autonomous) or mediated by extra-islet signals by perifusing isolated islets from *Slc7a2^–/–^* mice and *Slc7a2^+/+^* littermates with glucose, arginine, or a combination of the two. Basal glucagon secretion from *Slc7a2^–/–^* islets was low, even at low glucose, and arginine did not stimulate glucagon secretion from these isolated islets (Figure 3G). This appears to be due to a secretory defect in *Slc7a2^–/–^* α cells, as glucagon content was similar in *Slc7a2^+/+^* and *Slc7a2^–/–^* islets (Figure 3H), and membrane depolarization with KCl failed to stimulate glucagon secretion as it robustly did in *Slc7a2^+/+^* islets (Figure 3G). Arginine-stimulated insulin secretion was impaired in the *Slc7a2^–/–^* islets (Figure 3I), again, with no difference in insulin content (Figure 3J). KCl-stimulated and second phase glucose-stimulated insulin secretion was also impaired in *Slc7a2^–/–^* islets but not absent.

Taken together, these data indicate an islet-autonomous secretory defect in *Slc7a2^–/–^* α and β cells. Additionally, interrupted glucagon signaling does not influence the pattern of secretory profile in response to stimuli; rather, the genotype predicts the secretory response, including no response to the glucose/arginine bolus, in *Slc7a2^–/–^* mice (Supplemental Figure 3). However, the overall amount of glucagon was increased 5-fold in all genotypes by monoclonal antibody targeting the glucagon receptor (GCGR mAb) treatment versus IgG Ab–treated littermates, suggesting that glucagon secretion in *Slc7a2^–/–^* mice is blunted but not completely lost in response to other amino acids that are not transported by SLC7A2.

### Slc7a2^–/–^ mice have normal islet morphology and endocrine cell mass and islet-specific gene expression.

**Figure 4:**
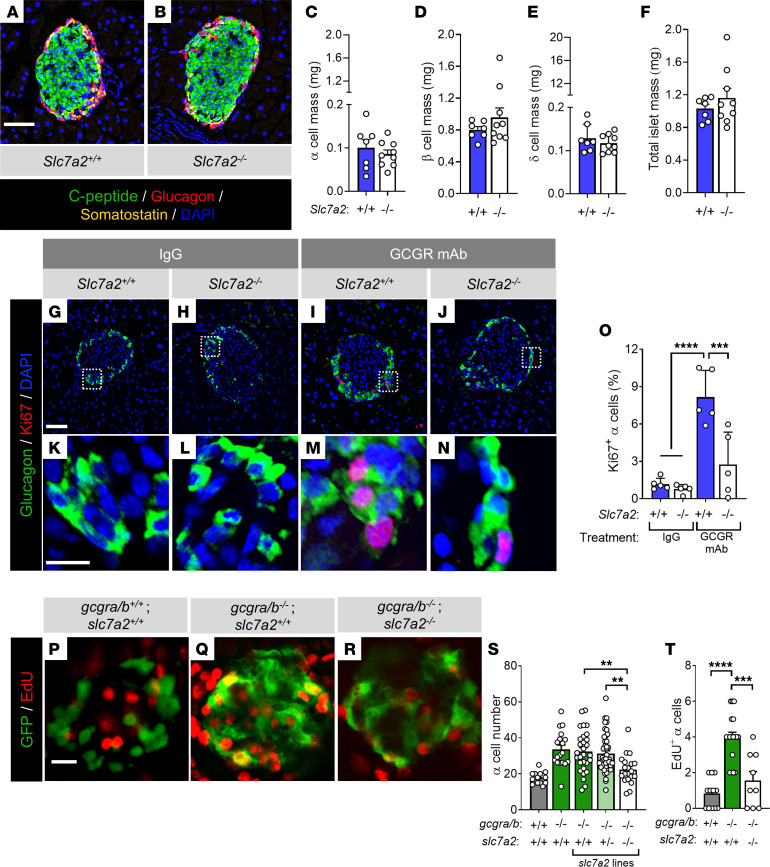
SLC7A2 is required for α cell proliferation in response to interrupted glucagon signaling. (**A** and **B**) Representative immunostaining for C-peptide, glucagon, and somatostatin from *Slc7a2^+/+^* and *Slc7a2^–/–^* mouse pancreas (scale bar = 50 mm). (**C**–**E**) Mass analysis for α (**C**), β (**D**), and δ (**E**) cells and total islet mass (**F**) from *Slc7a2^+/+^* (*n* = 7) and *Slc7a2^–/–^* (*n* = 9) mice. (**G**–**J**) Representative images of islets from *Slc7a2^+/+^* and *Slc7a2^–/–^* mouse pancreas after 2 weeks of treatment with GCGR mAb or control IgG (scale bar = 50 μm; inset **K**–**N** scale bar = 10 μm). (**O**) Quantification of α cell proliferation as determined by percent Ki67^+^/Gcg^+^ cells per total Gcg^+^ cells in *Slc7a2^+/+^* and *Slc7a2^–/–^* mouse islets after 2 weeks of treatment with GCGR mAb or control IgG (*n* = 5 each). (**P**–**R**) Representative images of 5 dpf islets from Tg(gcga:EGFP) zebrafish, α cell–specific EGFP, with CRISPR/Cas9-induced loss of glucagon receptors (gcgra/b) and/or *slc7a2* stained for EdU to assess proliferation (scale bar = 20 μm). (**S**) Quantification of total α cell numbers (*n* = 14, 18, 29, 46, and 20, respectively, for each genotype) and (**T**) quantification of EdU-positive α cells from zebrafish islets (*n* = 12, 18, and 9, respectively, for each genotype). **P < 0.005, ***P < 0.0005, and ****P < 0.0001.

*Slc7a2*^–/–^ mice have elevated SLC7A2-transported amino acids (e.g., arginine, lysine, and ornithine) (24). While these amino acids showed the greatest increases in *Slc7a2^–/–^* mice, we observed modest elevations in several other serum amino acids, including glutamine (Supplemental Figure 1J). To assess whether this slight elevation of amino acids, as compared with that of mice with interrupted glucagon signaling, is associated with increased α cell mass in the *Slc7a2^–/–^* mice, we performed immunofluorescence staining on pancreas sections from these mice. Islets from *Slc7a2^–/–^* mice had normal islet architecture and morphology (Figure 4, A and B) and no difference in α, β, or δ cell mass (Figure 4, C–F), similar to no difference being observed in the glucagon or insulin content of isolated islets (Figure 3, H and J).

### Interrupted glucagon signaling–stimulated α cell proliferation requires Slc7a2 expression.

Treatment of mice with a GCGR mAb interrupted glucagon signaling and stimulated α cell proliferation as previously shown (4, 9, 10). To investigate the role of SLC7A2 in α cell proliferation, we treated *Slc7a2^+/+^* and *Slc7a2^–/–^* with IgG Ab or GCGR mAb (Figure 4, G–O). In *Slc7a2^+/+^* mice, 2 weeks of treatment with GCGR mAb resulted in a greater than 6.8-fold increase in the percentage of Ki67-positive α cells (Figure 4, I, M, and O). *Slc7a2^–/–^* mice, treated in the same way, showed a 66% reduction in α cell proliferation (Figure 4, J, N, and O).

In a second model, we used a complementary zebrafish model of interrupted glucagon signaling to assess the role of *Slc7a2* expression in α cell proliferation. We previously described a *gcgr*-knockout zebrafish model with increased α cell proliferation and numbers (12). Deletion of *slc7a2* by CRISPR targeting in *gcgr*-null zebrafish decreased the total number of α cells and 5-ethynyl-2′-deoxyuridine–positive (EdU-positive) α cells at day 5 postfertilization (5 dpf) to wild-type fish levels, indicating that Slc7a2 is necessary for α cell proliferation in response to interrupted glucagon signaling in the developing zebrafish islet (Figure 4, P–T).

Together, these studies in mouse and zebrafish models of interrupted glucagon signaling indicate that SLC7A2 is necessary for α cell proliferation.

### SLC7A2 dependence of stimulated α cell proliferation is islet cell autonomous.

**Figure 5:**
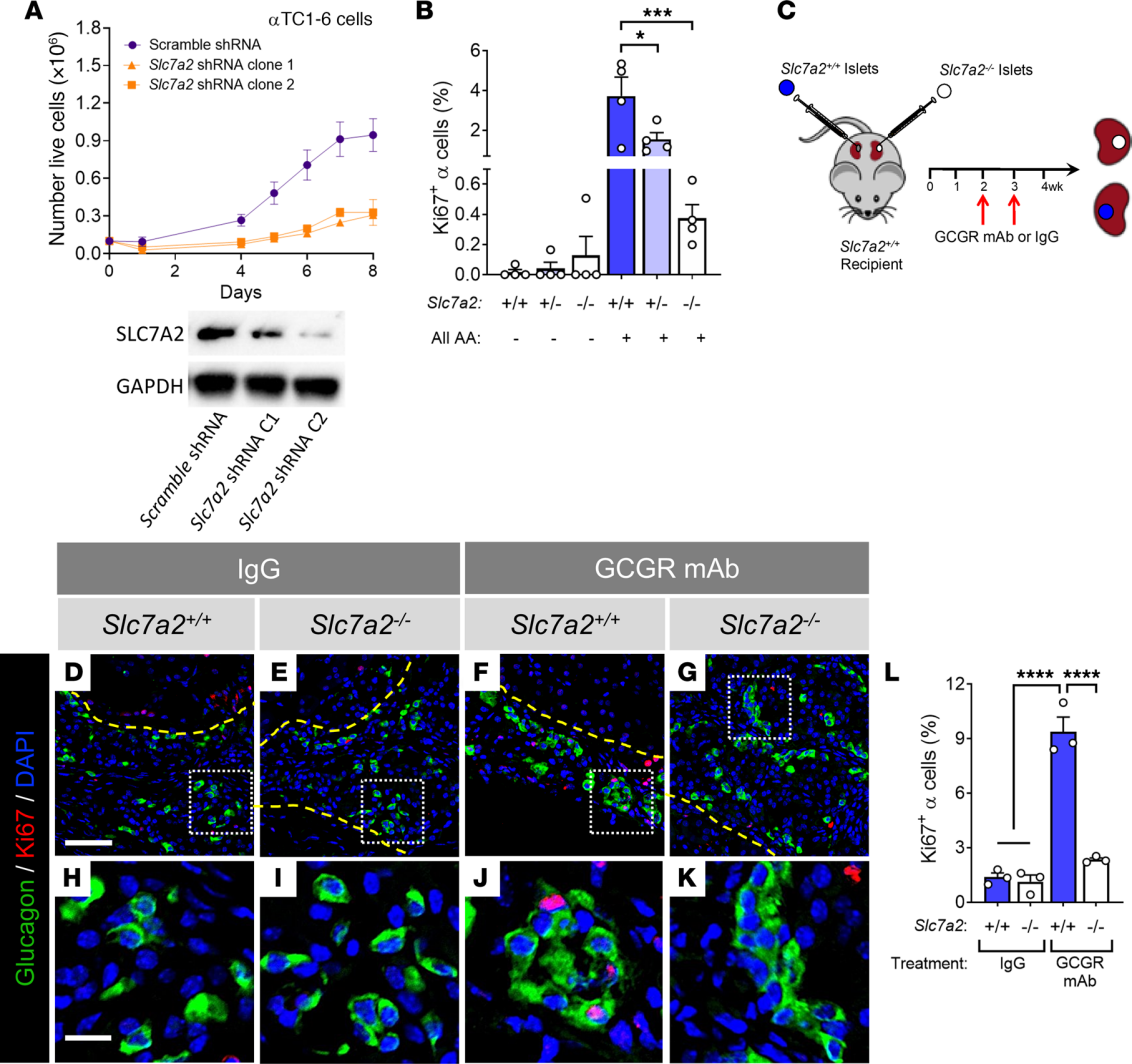
SLC7A2-dependent stimulated α cell proliferation is islet autonomous. (**A**) Cell growth over time of αTC1-6 cultured cells expressing *Slc7a2* shRNA (2 clones) or non-targeting (scrambled) control shRNA (*n* = 4 each). Immunoblot (below) shows decreased SLC7A2 protein in the *Slc7a2* shRNA lines. (**B**) Ex vivo α cell proliferation from isolated mouse islets cultured in low (–) and high (+) amino acid–containing medium as determined by percent Ki67^+^/Gcg^+^ cells per total Gcg^+^ cells in islets isolated from *Slc7a2^+/+^*, *Slc7a2^+/–^*, and *Slc7a2^–/–^* mice (*n* = 4 each). (**C**) Schematic of kidney capsule transplantation of *Slc7a2^–/–^* and *Slc7a2^+/+^* islets into *Slc7a2^+/+^* recipient mouse followed by glucagon receptor monoclonal antibody treatment. (**D**–**G**) Representative images of *Slc7a2^+/+^* and *Slc7a2^–/–^* islet grafts from *Slc7a2^+/+^* kidney capsules after 2 weeks of treatment with glucagon receptor monoclonal antibody (GCGR mAb) or control IgG (scale bar = 50 μm; inset **H**–**K** scale bar = 10 μm). Dashed yellow lines indicate kidney graft boundary. (**L**) Percent α cell proliferation from *Slc7a2^+/+^* and Slc7a2^–/–^ islets transplanted into *Slc7a2^+/+^* kidney capsule and treated with GCGR mAb or control IgG (*n* = 8 transplant recipients, *n* = 4 per treatment group). *P < 0.05, ***P < 0.0005, and ****P < 0.0001.

To evaluate whether reduced α cell proliferation in *Slc7a2^–/–^* mice is an islet-autonomous or an extra-islet effect, we first evaluated SLC7A2-dependent proliferation in clonal αTC1-6 shRNA cell lines. Two separately selected monoclonal *Slc7a2* shRNA lines showed decreased SLC7A2 protein as compared with a non-targeting (scrambled) shRNA–expressing line (Figure 5A). Evaluation of growth of these monoclonal lines over 8 days demonstrated that SLC7A2 loss reduced proliferation in these cells, with cell numbers approximately 65% lower in *Slc7a2* shRNA lines (Figure 5A). These data, when combined with the findings presented in Figure 1C, indicate that arginine and SLC7A2 are necessary for growth of the αTC1-6 mouse α cell line.

Isolated islets from *Slc7a2^–/–^* mice had a 90% reduction in α cell proliferation when cultured in high amino acids (Figure 5B). Interestingly, proliferation of *Slc7a2^+/–^* α cells was approximately 60% lower than *Slc7a2^+/+^*, suggesting that the level of *Slc7a2* expression may be rate limiting for proliferative response to arginine.

To test islet autonomy in vivo, we transplanted isolated islets from *Slc7a2^–/–^* mice into *Slc7a2^+/+^* recipients, placing the knockout islets into the physiological environment of a wild-type mouse. Each recipient mouse also had *Slc7a2^+/+^* islets transplanted into the contralateral kidney to serve as a control for stimulated α cell proliferation. After engraftment, recipient mice were treated weekly with either the GCGR mAb or the control IgG for an additional 2 weeks to investigate the consequence of interrupted glucagon signaling (Figure 5C). Immunostaining analysis of kidney grafts indicated that interrupted glucagon signaling increased α cell proliferation 4.5-fold in the *Slc7a2^+/+^* islet grafts, but α cell proliferation in *Slc7a2*^–/–^ grafts was not different from control, IgG treatment (Figure 5, D–L). Thus, these in vitro and in vivo data together indicate that SLC7A2 dependence of stimulated α cell proliferation is likely a result of SLC7A2 function in α cells.

### mTORC1 activity is required for arginine- and SLC7A2-dependent α cell proliferation.

**Figure 6:**
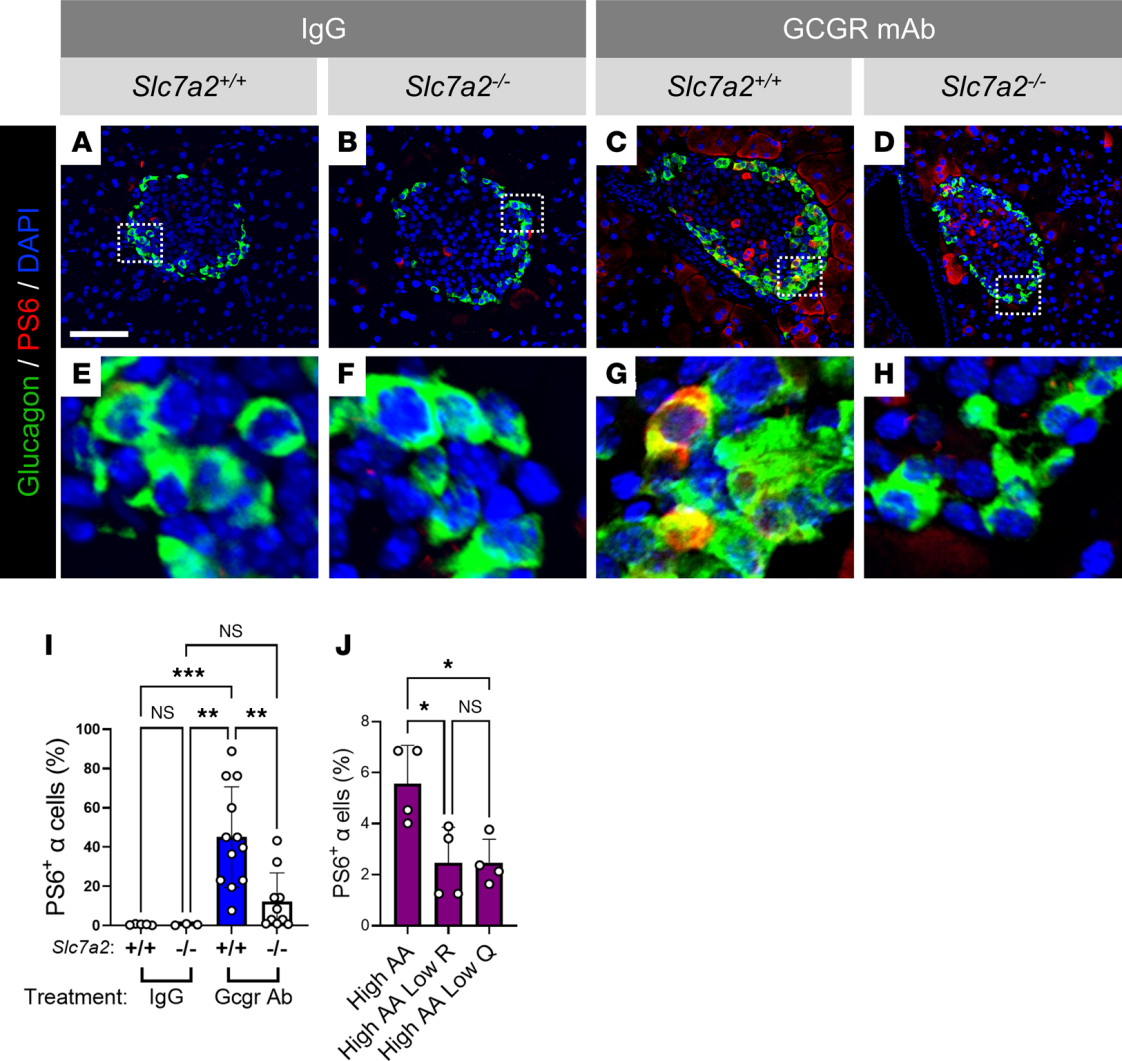
Arginine and SLC7A2 regulate mTORC1 activity. (**A**–**D**) Immunostaining of GCGR mAb– and IgG-treated *Slc7a2^+/+^* and *Slc7a2^–/–^* mouse islets for glucagon and phospho-ribosomal protein S6 (P-S6235/236), indicating active mTOR signaling (scale bar = 50 μm; inset **E**–**H** scale bar = 10 μm). (**I**) Quantification of α cell mTOR activation as determined by percent PS6^+^/Gcg^+^ cells (P-S6235/236) per total Gcg^+^ cells in *Slc7a2^+/+^* and *Slc7a2^–/–^* mouse islets after 2 weeks of treatment with GCGR mAb or control IgG (*n* = 4–12 each). (**J**) Quantification of α cell mTOR activation as determined by percent PS6^+^/Gcg^+^ cells per total Gcg^+^ cells in *Slc7a2^+/+^* and *Slc7a2^–/–^* cytospun dispersed mouse islets after 4-day culture in high amino acids, high amino acids with low arginine (R), or high amino acids with low glutamine (Q) (*n* = 4). *P < 0.05, **P < 0.005, ***P < 0.0005.

Stimulated α cell proliferation has been mechanistically linked to mTOR complex 1 (mTORC1) signaling and increased phosphorylation of ribosomal protein S6 (phospho-S6), a target of mTORC1 (10). Glutamine has previously been shown to regulate α cell proliferation in an mTOR-dependent manner (10); however, absence of glutamine alone does not fully eradicate the proliferative effect of glucagon receptor–antagonized wild-type mice. Therefore, to assess whether arginine transport through SLC7A2 could affect mTORC1 activity in α cells, we quantified phospho-S6 expression by immunostaining pancreas sections from wild-type or *Slc7a2*^–/–^ mice. We observed reduced phospho-S6 expression levels in α cells of GCGR mAb–treated *Slc7a2^–/–^* mice to similar levels as IgG-treated wild-type and *Slc7a2*^–/–^ mice when compared with GCGR mAb–treated wild-type mice. This suggests that the inactivation of the SLC7A2 transporter can reduce mTOR activity in glucagon receptor–antagonized mice (Figure 6, A–I, and Supplemental Figure 4, A–D). To determine if arginine can similarly regulate mTORC1 activity, mouse islets were isolated and dispersed into single cells, cultured in high amino acid conditions or high amino acid conditions with low glutamine or arginine concentrations, followed by phospho-S6 immunofluorescence (Figure 6J). We observed a 2.2-fold reduction in phospho-S6^235/236^ expression in the high amino acid conditions with either low arginine or low glutamine, suggesting that removal of arginine or glutamine can reduce mTORC1 activation correspondingly. The data insinuate that arginine and SLC7A2 may regulate α cell proliferation in an mTORC1-dependent manner.

### Stimulated Slc38a5 expression during interrupted glucagon signaling is SLC7A2 dependent.

**Figure 7:**
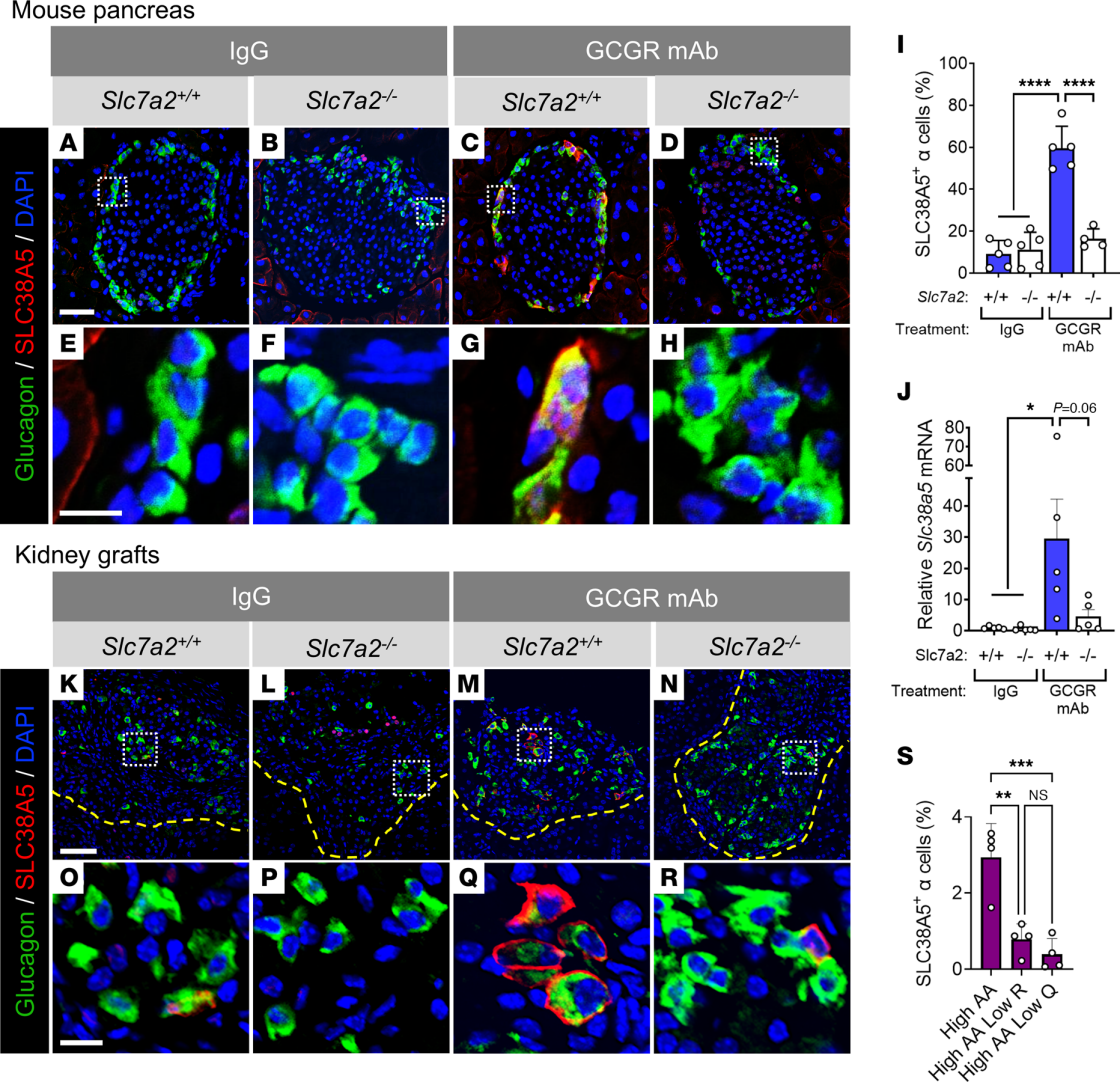
SLC7A2 is required for upregulation of Slc38a5 expression during interrupted glucagon signaling. (**A**–**D**) Representative images of islets from *Slc7a2^+/+^* and *Slc7a2^–/–^* mouse pancreas stained for glucagon (green) and SLC38A5 (red) after 2 weeks of treatment with GCGR mAb or control IgG (scale bar = 50 μm; inset **E**–**H** scale bar = 10 μm). (**I**) Quantification of SLC38A5 expression in α cells by percent SLC38A5^+^/GCG^+^ cells per total Gcg^+^ cells (% SLC38A5^+^ α cells) in *Slc7a2^+/+^* and *Slc7a2^–/–^* mouse islets after injection with GCGR mAb or control IgG (*n* = 4–5). (**J**) Quantification of *Slc38a5* mRNA levels assessed by quantitative PCR in *Slc7a2^+/+^* and *Slc7a2^–/–^* mice treated with IgG or GCGR mAb. (**K**–**N**) Representative images of *Slc7a2^+/+^* and *Slc7a2^–/–^* islet grafts from *Slc7a2^+/+^* kidney capsules after 2 weeks of treatment with GCGR mAb or control IgG and stained for glucagon and SLC38A5 (scale bar = 50 μm; inset **O**–**R** scale bar = 10 μm). Dashed yellow lines indicate kidney graft boundary. (**S**) Quantification of SLC38A5 in α cells as determined by percent SLC8A5^+^/Gcg^+^ cells per total Gcg^+^ cells in wild-type mouse islets after 4-day culture in high amino acid–containing medium (High AA) or in otherwise high AA medium with low arginine (Low R) or low glutamine (Low Q). *P < 0.05, **P < 0.005, ***P < 0.0005, ****P < 0.00005.

**Figure 8:**
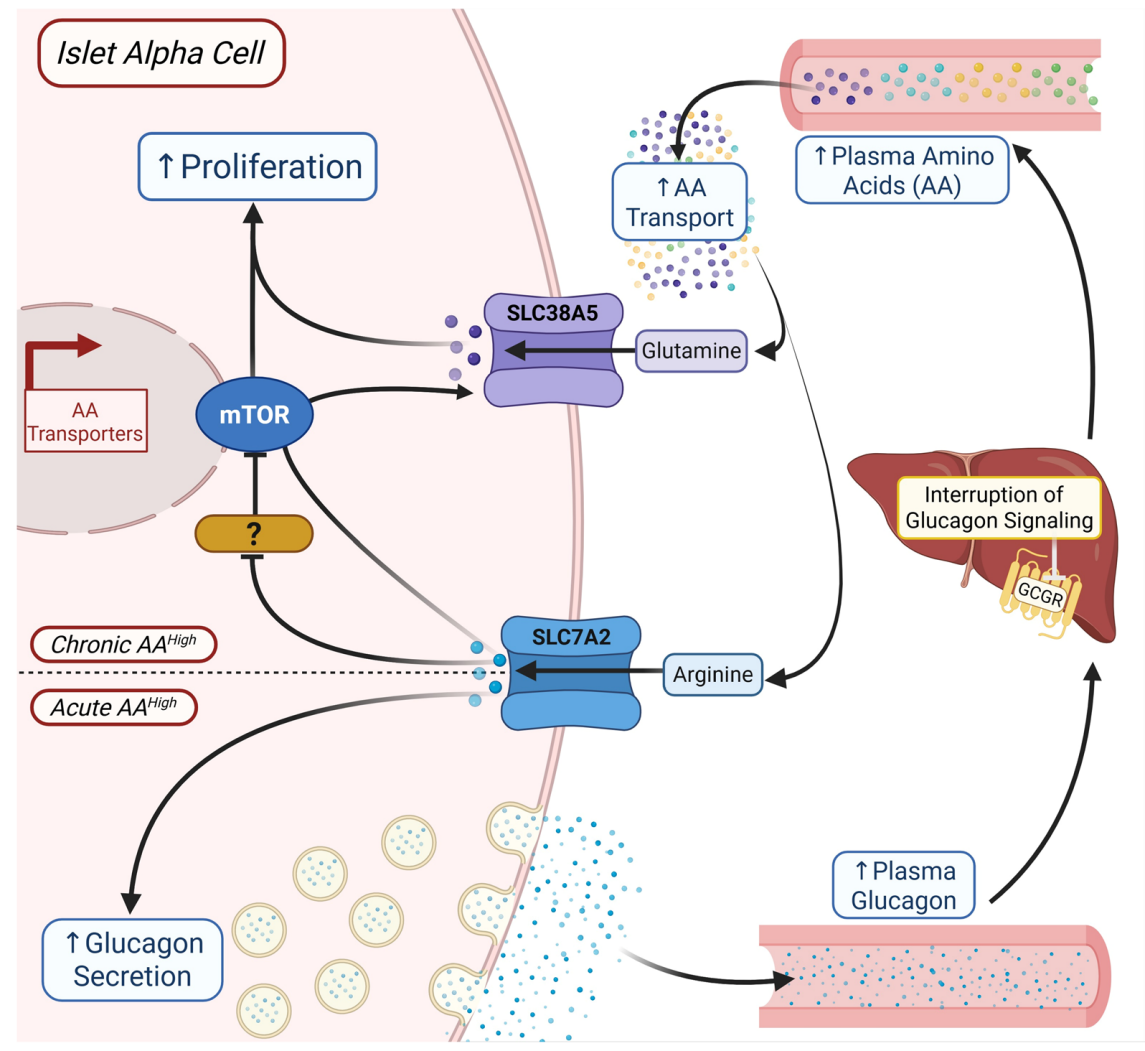
Model of α cell response to elevated amino acids during interrupted glucagon signaling. SLC7A2 is required for arginine-stimulated glucagon secretion and α cell proliferation. Arginine transported into the α cell through SLC7A2 initially stimulates glucagon secretion (Acute AAHigh). The buildup of intracellular arginine ultimately stimulates mTOR-dependent Slc38a5 expression through metabolism or other physiological mechanisms (Chronic AAHigh). Transport of glutamine through SLC38A5 stimulates α cell proliferation as described previously (9, 10). Created with BioRender.com.

Previous studies showed that interrupted glucagon signaling stimulates expression of *Slc38a5*, a neutral amino acid/glutamine transporter, in mouse and zebrafish α cells, and that this is partially required for mTOR-dependent α cell proliferation in an elevated amino acid environment associated with interrupted glucagon signaling in liver (9, 10). To test whether *Slc38a5* expression was SLC7A2 dependent, we evaluated the presence of SLC38A5 in α cells of GCGR mAb–treated *Slc7a2^–/–^* mice and *Slc7a2^+/+^* littermates by immunofluorescence (Figure 7, A–I). As expected, SLC38A5 was detected in greater than 60% of GCGR mAb–treated *Slc7a2^+/+^* α cells versus 10% in IgG-treated littermates. Interestingly, less than 20% of *Slc7a2^–/–^* α cells showed detectable SLC38A5 similar to IgG-treated mice of either genotype (Figure 7, A–I). Isolated *Slc7a2^–/–^* islets transplanted into the kidney capsules of *Slc7a2^+/+^* mice subsequently treated with GCGR mAb also lacked this stimulated SLC38A5 expression (Figure 7, K–R). Isolated islets from *Slc7a2^–/–^* mice treated with GCGR mAb also had impaired induction of *Slc38a5* gene expression (Figure 7J). To further assess the connection between SLC7A2 and SLC38A5, mouse islets were isolated and dispersed into single cells, cultured in high amino acid conditions, or high amino acid conditions with low glutamine or arginine concentrations, followed by SLC38A5 immunofluorescence (Figure 7S). SLC38A5^+^ α cells were reduced in conditions with high amino acid concentrations coupled with either low glutamine or low arginine concentrations. No significant differences were found between low arginine and low glutamine exposure, indicating arginine and glutamine are equally important for the induction of the glutamine transporter SLC38A5. Overall, these data suggest that arginine transport through SLC7A2 is required for the induced gene expression of the glutamine transporter SLC38A5 observed during interrupted glucagon signaling (Figure 8).

## Discussion

Arginine is a major substrate for SLC7A2 transport and a known potent secretagogue for both pancreatic islet α and β cells. We found that *SLC7A2*, one of the most highly expressed amino acid transporter genes in α cells in mice and humans, plays a central role in islet function. Loss of *Slc7a2* expression in mice resulted in impaired arginine-stimulated glucagon and insulin secretion in vivo and ex vivo. *Slc7a2* expression is also required for amino acid–stimulated α cell proliferation in zebrafish and mice. Together, our data indicate that SLC7A2 is the primary arginine transporter in islet cells regulating arginine’s effects on hormone secretion. Our work also provides strong evidence that arginine stimulation of hormone secretion works by an alternative mechanism than what has been previously suggested (37). These studies also directly link SLC7A2 function with the previous observation that glutamine transport through SLC38A5 is necessary for α cell proliferation by demonstrating that arginine transport through SLC7A2 is required for mTOR activation and upregulation of *Slc38a5* expression (see model, Figure 8). Finally, we show that SNPs in the SLC7A2 gene are associated with HbA1c, a common clinical value used to evaluate glycemic status. These roles for SLC7A2 in α cells described here support its designation as an α cell “signature gene” (38).

Previous studies suggested that arginine transport results in membrane depolarization due to the cationic properties of arginine (39). Yet, glucagon secretion from *Slc7a2^–/–^* islets was not stimulated in perifusion experiments even with the strong depolarizing agent KCl, while glucagon content and overall α cell mass were not different (Figure 3G). This suggests that impaired hormone secretion when SLC7A2 expression is lost represents a fundamental defect in secretory mechanisms or machinery versus secretory capacity and not simply perturbations in membrane polarity. Yet, islet transcriptomics studies in *Slc7a2^–/–^* and *Slc7a2^+/+^* islets did not reveal any insight into a possible mechanism (e.g., changes in ion channel or vesicle machinery expression; Supplemental Figure 2). Furthermore, there remains uncertainty as to whether the observed insulin secretion defect is β cell autonomous or the result of impaired paracrine signaling from α cells. Interestingly, early studies with the perfused rat pancreas showed that arginine rapidly and potently stimulates glucagon secretion under no glucose conditions whereas its effect on insulin secretion is slow and tonic, requiring several minutes for appreciable insulin secretion. This suggests that SLC7A2 transport levels limit the response by islet cells to arginine (40) similar to the intermediate effect of an arginine bolus on insulin levels in *Slc7a2^+/–^* mice versus *Slc7a2^–/–^* mice observed in this study. However, under high-glucose conditions, both glucagon and insulin are rapidly and potently secreted in response to arginine. This indicates that factors other than SLC7A2 transporter levels may limit β cell responses to arginine. The glucose dependency observed in this study is similar to responses observed with incretin-stimulated insulin secretion where incretins have little effect on insulin secretion under low glycemic or euglycemic conditions. Intra-islet glucagon signaling through both glucagon and GLP-1 receptor signaling in β cells required for arginine-stimulated insulin secretion (40, 41). We observed a modest decrease in glucose-stimulated insulin secretion in *Slc7a2^–/–^* islets, while arginine-stimulated insulin secretion was profoundly perturbed, in addition to a loss of glucagon secretion. Therefore, we predict that much of arginine’s effect on β cells might be paracrine in nature, mediated via proglucagon-derived peptides linked to the α cell’s robust expression of SLC7A2. To understand whether the loss of arginine-stimulated insulin secretion in *Slc7a2^–/–^* mice results from the intrinsic loss of SLC7A2 from β cells or whether it is the result of the observed loss of glucagon secretion, new models are necessary (a pseudoislet system composed of mixing *Slc7a2^–/–^* α cells with *Slc7a2^+/+^* β cells or newly generated α and β cell–specific *Slc7a2*-knockout mice). Therefore, further study is needed to determine how arginine and SLC7A2 promote secretion.

Human islets have been shown to have a splicing quantitative trait locus for the *SLC7A2* gene, which are known to regulate alternative splice events of pre-mRNA (42). Thus, the SNPs within this region could result in altered splicing products from the gene locus. *SLC7A2/Slc7a2* is alternatively spliced at exon 7, yielding 2 protein isoforms, CAT2A and CAT2B (20). While both exons appear to be expressed in α and β cells, α cells predominately express the second exon 7 associated with SLC7A2A (CAT2A) isoform, and β cells prefer the first exon 7 associated with SLC7A2B (CAT2B). This suggests that α cells have higher levels of CAT2A, a low-affinity (*K_m_* 2–5 mM), high-capacity arginine transporter. This difference, in addition to overall higher expression, might grant α cells the ability to sense rising levels of arginine better than other islet cells. Our analysis of the association to HbA1c levels in the EXTEND GWAS dataset suggests that individuals with either of 2 SNPs within the first intron of SLC7A2 have reduced HbA1c levels (i.e., negative β values). It is unclear if this association is due to glycemia, as only a third of variance in HbA1c levels in non-diabetic individuals is due to factors such as glycemia and body mass index (42). Interestingly, *SLC7A2* is downregulated in both T1D and T2D human α cells and may reflect observed downregulation of *MAFB*/MAFB under both conditions (25, 43–45). In mouse islets, *Slc7a2* expression is positively correlated with the expression of *Foxa2* and *Mafb* (46). That these SNPs are proximal to binding sites for MAFB and FOXA2 in human islets suggests that they may influence their binding and subsequent regulation of *SLC7A2* expression. However, it is unclear where SLC7A2 expression is driving such associations, since MAFB is expressed in both human α and β cells and FOXA2 is expressed in both α cells and liver. Despite associations of SNPs in SLC7A2 with improved long-term glucose homeostasis in humans, *Slc7a2^–/–^* mice had normal fasting glucose and glucose tolerance when assessed by acute intraperitoneal glucose injection. To test if the incretin system affected blood glucose levels in the absence of SLC7A2 transport, we performed an oral mixed meal tolerance test (Supplemental Figure 1, D–F). We observed no changes in glucose tolerance even with an activated incretin system. However, our study supports that arginine regulation of hormone secretion itself may be the driver behind the association and not glucose disposal per se.

Our work connects arginine signaling to glutamine signaling by showing *Slc7a2^–/–^* mice lack the increased expression of the glutamine transporter, *Slc38a5*, seen in *Slc7a2^+/+^* α cells, and described in previous studies (9, 10), when glucagon signaling is interrupted. SLC38A5 is associated with mTOR-mediated increased proliferation (10), which we show is SLC7A2 dependent. In addition to amino acid–dependent mTORC1 signaling (Figure 6), others and we have shown that additional signaling pathways through the calcium sensor receptor, ERK, and ERBB3 growth factor are required for α cell proliferation (47–51). Together, these findings suggest a potential connection between SLC7A2- and mTOR-mediated α cell proliferation.

Cancer cells also upregulate amino acid transport and metabolic pathways to facilitate their growth but under nutrient-limiting conditions. In cancer cells, arginine-dependent regulation of cytosolic CASTOR1 and lysosomal SLC38A9 and TM4SF5 proteins promotes mTOR activation leading to proliferation (52). Whether these pathways for arginine-stimulated proliferation are conserved in non-transformed cells including α cells is unclear. In contrast with the present study, SLC7A2 expression is negatively correlated with proliferation in breast cancer, colon cancer, hepatocellular carcinoma, and ovarian cancer (53–56). SLC7A2 is highly expressed in multiple normal tissues, including liver, breast, skeletal muscle, and islets. Thus, the role of SLC7A2 in cell proliferation and physiology in general merits cell type–specific targeting for further study.

It was surprising that no difference in α cell mass was observed in untreated *Slc7a2^–/–^* mice, since SLC7A2 was required for adaptive growth in response to hyperaminoacidemia and glucagon secretion was nearly absent under our conditions. This suggests that other mechanisms might also determine baseline α cell mass. However, we predict that there is sufficient glucagon signal in the liver in these mice to not fully activate the liver/α cell axis as with pharmacological or genetic targeting glucagon receptors. This hypothesis is supported by the observation that while *Slc7a2^–/–^* mice have mild hyperaminoacidemia, blood levels of amino acids such as glutamine and arginine are 3- to 5-fold lower than levels observed in mice with interrupted glucagon signaling (9–11). Expression of *Slc38a5* is greatly increased in α cells during interrupted glucagon signaling, and it is the only amino acid transporter that responds as such. Interestingly, *Slc38a5^–/–^* mice showed only 50% lower α cell proliferation during interrupted glucagon signaling than wild-type mice, indicating that other amino acids or transporters are required for the α cell proliferative response (9). These observations led us to hypothesize that other amino acids may play an essential role in α cell proliferation.

Arginine plays several critical physiological roles, including controlling vasodilation through the synthesis of nitric oxide, immune function, and removing ammonia from the body via production of urea. Glucagon facilitates ureagenesis by both transcriptional and posttranslational control of the urea cycle in liver. Conversely, several amino acids, including arginine, potently stimulate glucagon secretion. Together, these events form an endocrine feedback loop called the liver/α cell axis, where α cells play a critical role in the sensing of circulating amino acids (Figure 8) (1, 57). When arginine levels rise acutely (e.g., minutes to hours), such as with a protein meal, arginine transport via SLC7A2 leads to glucagon secretion. Similarly, chronic hyperargininemia (e.g., days or longer), such as with interruption of glucagon signaling in liver, and prolonged arginine accumulation via SLC7A2 lead to mTOR activation in α cells and activation of gene expression (including other amino acid/glutamine transporters, such as *Slc38a5*) that facilitate cell proliferation.

The discovery that interrupted glucagon signaling stimulates human α cell proliferation (4, 14) and that human α cells can transdifferentiate into β cells (58) holds promise for the use of glucagon receptor antagonists for the reestablishment of β cell mass after diabetic loss as has been recently demonstrated in mice (4). Safely expanding α cells could also represent the first step in restoring β cell mass through α-to-β cell transdifferentiation. Further studies are needed to address the molecular mechanisms by which arginine stimulates glucagon secretion and arginine interacts with the mTOR pathway in α cells to stimulate *Slc38a5* expression and proliferation, whether transport of other amino acids is required for these responses, and whether these mechanisms are conserved in human islets.

## Methods

Further information can be found in Supplemental Methods.

### Sex as biological variable.

Both male and female mice were combined in these studies, as there were no differences in response to intervention observed between sexes.

### Mouse studies.

All mouse studies were performed at Vanderbilt University Medical Center and approved by the Institutional Animal Care and Use Committee. Mice were housed on a 12-hour light/dark cycle with ad libitum access to standard rodent chow (unless indicated for fasting purposes) and water. *Slc7a2^–/–^* mice (Jackson Laboratory, B6.129S7-*Slc7a2^tm1Clm^*/LellJ) and *Slc7a2^+/–^* and *Slc7a2^+/+^* littermates from heterozygous crosses were used for all mouse experiments. To interrupt glucagon signaling, mice were treated weekly with 10 mg/kg of a humanized monoclonal antibody targeting the glucagon receptor (GCGR mAb “Ab-4”) intraperitoneally once a week for 2 weeks (59).

For contralateral islet transplantations, 100–150 islets isolated from 14- to 16-week-old *Slc7a2^–/–^* donor mice were transplanted under the left kidney capsule, and an equivalent number of *Slc7a2^+/+^* donor islets were transplanted under the right kidney capsule of an *Slc7a2^+/+^* recipient mouse. Two weeks after transplantation, transplant recipients were given 2 weekly treatments of GCGR mAb as described in Figure 5C and above. After 2 weeks of GCGR mAb treatment, kidneys containing grafts were retrieved, bisected at the grafts, fixed in 4% paraformaldehyde, embedded in OCT (Thermo Fisher Scientific), and stored at –80°C until sectioning (14, 60).

### Zebrafish studies.

The role of Slc7a2 in proliferation of α cells in zebrafish (*Danio rerio*) was studied using a previously described (12) glucagon receptor double-knockout line expressing GFP under the control of the glucagon promoter to identify α cells: *Tg(gcga:GFP)*;*gcgra/b^–/–^*. Using CRISPR/Cas9 technology, a 62 bp deletion was created in *slc7a2* (using the sgRNA GGGTAAGCGCCAGTCGCCAG and the PAM TGG for targeting). We assessed total α cells by counting GFP^+^ cells in the islets of 5 dpf fish as described previously (12). To identify proliferating α cells, embryos were incubated with 1 mmol/L EdU at 4 dpf and chased for 24 hours. EdU was detected according to published protocols (12) and using the Click-iT EdU Alexa Fluor 594 Imaging Kit (C10339; Invitrogen). All images were collected using a Zeiss LSM880 confocal microscope (Carl Zeiss).

### Tissue collections from mice.

After treatment periods, pancreata were collected. Histology samples were fixed in 4% paraformaldehyde, embedded in OCT, and stored at –80°C until use. For transplantation, islet RNA, and ex vivo proliferation experiments, islets were isolated by intraductal infusion of collagenase P and histopaque gradient separation (both MilliporeSigma).

### Stimulated glucagon and insulin secretion in vivo.

To assess the role of SLC7A2 in glucagon and insulin secretion, *Slc7a2^+/+^*, *Slc7a2^+/–^*, and *Slc7a2^–/–^* mice were fasted for 6 hours and then injected intraperitoneally with glucose, arginine, or both to final concentrations of 2 g/kg body weight each. Blood was collected retroorbitally before injection (fasting) and 15 minutes after injection (stimulated). Blood glucose was measured with a handheld glucometer (Accu-Check Aviva), and remaining whole blood was spun. Serum was collected into separate tubes and stored at –80°C for glucagon and insulin analyses.

Serum hormones were analyzed in the Vanderbilt Hormone and Analytical Services core. Serum glucagon was analyzed in a 2-site enzyme sandwich ELISA (Mercodia). Serum insulin was analyzed by dual antibody radioimmunoassay and counted in a Packard Gamma counter.

### Immunofluorescence staining and image analysis.

To compare islet cell masses in untreated *Slc7a2^+/+^* and *Slc7a2^–/–^* mice, pancreata from 14- to 16-week-old mice were prepared for histology as described above. Whole mouse pancreas was sectioned on a cryostat at a thickness of 8 mm per section. The full depth of the pancreas was sectioned by 7 repeated steps of sectioning away 150 mm and collecting 10 sections onto slides. In this way 7 depths of 150 mm were collected from each pancreas. For islet cell mass analysis, 1 section from each of the 7 depths was stained for C-peptide (Invitrogen, PA-85595) to mark β cells, glucagon (LSBio, LS-C202759) to mark α cells, and somatostatin (Santa Cruz Biotechnology, sc-7819) to mark δ cells. Whole pancreatic sections were imaged on Scanscope FL System (Aperio Technologies), and islet cell areas were analyzed using Halo image analysis software (Indica Labs). Total pancreatic islet cell masses were calculated as described previously (61). Briefly, islet cell areas from each of 7 depths were normalized to total pancreas section area, areas from each of the 7 sections were summed, and the sum was multiplied by total pancreas mass to achieve an estimate of the percentage of total mass.

For α cell proliferation analysis, pancreata from mice treated for 2 weeks with GCGR mAb were sectioned and immunostained for the proliferation marker, Ki67 (Abcam, ab15580), and glucagon to mark α cells. Whole sections were imaged on the Scanscope and analyzed on Halo software. Percent α cell proliferation was calculated from at least 1,000 α cells per animal by dividing Ki76^+^/glucagon^+^ cells by total glucagon^+^ cells. Similarly, percent *Slc38a5^+^* α cells was calculated by staining for *Slc38a5* (Santa Cruz Biotechnology, sc-50682) and glucagon, with imaging and analyzing as above. Amino acid–stimulated α cell proliferation has been shown to be mTOR dependent. We verified this mTOR dependence by staining for a target of mTOR1 activity, phosphorylation of ribosomal protein S6 (Cell Signaling Technology: pS6^235/236^, 4858S; pS6^240/244^, 5364S), and glucagon.

Kidney grafts were sectioned at 5 mm per section, and approximately 20 sections were collected for each graft. These sections were immunostained for glucagon and Ki67 to assess percent α cell proliferation from at least 500 α cells per graft. Grafts were also stained for glucagon and SLC38A5 to assess α cell–specific expression of the transporter during interrupted glucagon signaling.

### Ex vivo α cell proliferation.

Ex vivo α cell proliferation was assessed as described previously (10). Briefly, islets isolated from *Slc7a2^–/–^*, *Slc7a2^+/–^*, and *Slc7a2^+/+^* mice were cultured in DMEM-based medium with high or low amino acid concentrations, based on amino acid levels in *Gcgr^–/–^* and *Gcgr^+/+^* mouse serum, respectively (see Supplemental Table 1 for amino acid concentrations in islet culture media), for 72 hours. See Figure 1, A and B, for ex vivo α cell proliferation analysis.

After culture, islets were washed in 2 mM EDTA and dispersed with 0.025% trypsin at 37°C for 10 minutes with mixing. Dispersed islet cells were recovered by centrifugation at 1,000*g* in RPMI media containing 5.6 mM glucose, 10% FBS, and 1% Penicillin/Streptomycin. The resulting cell pellet was resuspended in medium and centrifuged onto a glass slide using a Cytospin 4 (Thermo Fisher Scientific) centrifuge. We used 800 rpm for 3 minutes. Slides were air-dried for 30 minutes, then stored at –80°C until use. For staining, slides were thawed, immediately fixed in 4% paraformaldehyde, and immunostained for glucagon, to mark α cells, and Ki67, to mark proliferating cells. Slides were imaged using a Leica Microsystems Epifluorescent Microscope DM1 6000B. The percent of proliferating α cells was calculated based on the number of Ki67^+^/glucagon^+^ cells divided by the total number of glucagon^+^ cells using Imaris image analysis software (Oxford Instruments).

### αTC1-6 cell culture.

αTC1 clone 6 (αTC1-6) cells were obtained from ATCC (CRL-2934) and maintained in DMEM, low glucose (Gibco 11885-084), with 10% FBS, 15 mM HEPES, 0.1 mM non-essential amino acids, 0.02% bovine serum albumin, and 1% Penicillin/Streptomycin (Supplemental Table 2). To test the roles for arginine and glutamine in growth of these cells, SILAC DMEM Flex Media (Gibco, A24939-01), which lacks arginine, glutamine, and lysine, was used as base medium, prepared as above, and supplemented with lysine to produce No Gln / No Arg control medium. The control medium was supplemented with 0.4 mM arginine (No Gln / 0.4 mM Arg) or 4 mM glutamine (4 mM Gln / No Arg) or both (4 mM Gln / 0.4 mM Arg) to test the effects of glutamine and arginine on αTC1-6 cell growth. To assess cell growth, 6-well plates were seeded with 1 × 10^5^ cells/well (day 0) in standard culture medium and allowed to normalize overnight. Cells from 1 well in each 6-well plate were collected and counted (day 1) in a Countess 3 automated cell counter (Thermo Fisher Scientific). Media were changed in the remaining wells to experimental media described above, and cells were allowed to grow for 3 days. Beginning on day 4, cells from 1 well in each media condition were collected and counted, and growth curves were established (Figure 1C).

To establish the requirement for SLC7A2 in αTC1-6 cell growth, monoclonal lines expressing *Slc7a2* shRNA or a scrambled (non-targeting) shRNA control were established. Cells were transduced with lentivirus expressing *Slc7a2* shRNA (VectorBuilder vector no. VB170727-1121dxf) or scrambled shRNA (VectorBuilder vector no. VB170313-1108xyf), each expressing a puromycin resistance cassette and a GFP fluorescence marker. Transduced cells were selected with 2.5 mg/mL Puromycin Dihydrochloride (Corning, 61-385-RA) to produce polyclonal lines expressing these shRNAs. Polyclonal lines were seeded at a density of 1 cell per well in 96-well plates and allowed to grow into single colonies in each well. Colonies were expanded and selected based on *Slc7a2* expression (Figure 5A, Western blot). Cell growth of these monoclonal shRNA lines was assessed as described above in basal DMEM-based medium.

### Analysis of published RNA-seq datasets.

Normalized RNA-seq data were obtained from the National Center for Biotechnology Information (NCBI) Gene Expression Omnibus (GEO) repository. Published datasets for sorted human (25, 26), mouse (27), and zebrafish (28) α and β cells were selected for comparison of transporter expression between the 2 cell types. Human and zebrafish expression data were normalized with TMM method, and mouse data were normalized to RPKM. Normalized expression data for all solute carriers (SLC genes) in α and β cells were sorted from highest to lowest expression, and the top 50 in each cell type are given in Supplemental Table 3. The 5 most highly expressed cationic amino acid transporters in α cells, sorted from highest to lowest, are shown for all 3 species in Figure 1, D–F.

### Whole islet RT-PCR.

For RT-PCR analyses, *Slc7a2^+/+^* and *Slc7a2^–/–^* mice were treated with IgG or GCGR mAb or untreated. Islets were isolated as described above. RNA was isolated from whole islets. Trace DNA was removed with the RNAqueous micro total RNA isolation kit (Thermo Fisher Scientific). RNA integrity was evaluated by Agilent 2100 Bioanalyzer. cDNA was synthesized from high-integrity (RIN > 7) total RNA using HighCapacity cDNA Reverse Transcription Kit (Applied Biosystems) according to the manufacturer’s instructions. mRNA levels were assessed by quantitative PCR using the TaqMan assay system. Deletion of *Slc7a2* was validated by exon 2–specific quantitative real-time RT-PCR on RNA isolated from *Slc7a2^–/–^* and wild-type littermate (*Slc7a2^+/+^*) islets (Supplemental Figure 2B). Primers were purchased from Thermo Fisher Scientific: *Actb* (internal control), Mm02619580_g1; *Slc7a2*, Mm00432032_m1; and *Slc38a5*, Mm00549967_m1.

### In vitro islet perifusion.

Function of isolated *Slc7a2^+/+^* and *Slc7a2^–/–^* islets was studied in a dynamic cell perifusion system at a perifusate flow rate of 1 mL/min as described previously (25, 62). Stimulus concentrations and exposure times are shown in Figure 3 traces. The effluent was collected at 3-minute intervals using an automatic fraction collector. Glucagon and insulin concentrations in each perifusion fraction and islet extracts were measured by radioimmunoassay (MilliporeSigma).

### Statistics.

All data are shown with error bars indicating standard error of the mean. Data within individual experiments were compared with ordinary 1-way ANOVA using Tukey’s correction for multiple comparisons, unless otherwise designated in the figure legend. *P* < 0.05 was considered statistically significant.

### Study approval.

All mouse studies were performed at Vanderbilt University Medical Center and approved by the Institutional Animal Care and Use Committee.

### Data availability.

All RNA-seq data are from previously published studies and available in public repositories as indicated above. Any additional data are in the Supporting Data Values file or available from the corresponding author upon request.

## Author contributions

EDD, WC, and ACP contributed to conceptualization. WC, ACP, EDD, MPK, KWS, JES, and ADA contributed to funding acquisition. ES, EDD, JES, MS, WS, CD, AB, KWS, MPK, GP, LY, RJ, KS, KCC, SS, AMRS, TS, MW, KTW, and XL contributed to investigation. WC, ACP, and EDD contributed to supervision. ES and EDD wrote the original draft. ES, EDD, JES, ACP, WC, MPK, AMRS, KCC, and KWS contributed to writing – review & editing. All authors approved the final manuscript.

## Conflict of interest

KWS is an employee of Eli Lilly & Co.

## Funding support

This work is the result of NIH funding, in whole or in part, and is subject to the NIH Public Access Policy. Through acceptance of this federal funding, the NIH has been given a right to make the work publicly available in PubMed Central.

- Breakthrough T1D SRA-149-Q-R (to ACP and EDD).
- NIH R01DK117147 (to WC and ACP).
- NIH R01DK132669 (to EDD).
- American Heart Association 24TPA1278431 (to EDD).
- NIH F31DK134158 (to JES).
- NIH T32DK007563 (to JES and AMRS).
- NIH R25GM134979 (to TS).
- NIH K01DK117969 (to EDD).
- University of Wisconsin–Madison, Department of Biochemistry and Office of the Vice Chancellor for Research and Graduate Education with funding from the Wisconsin Alumni Research Foundation (MPK).
- NIH grant R01DK101573 (to ADA).
- NIH grant R01DK102948 (to ADA).
- NIH grant RC2DK125961 (to ADA).
- NIH grant R01DK128200 (to KTW).
- NIDDK-supported Human Islet Research Network (UC4 DK104211 and DK112232).
- NIH DK106755 (to ACP).
- VUMC’s Digestive Diseases Research Center (P30 DK058404).
- Department of Veterans Affairs BX000666.
- Department of Veterans Affairs I01CX002171.
- Vanderbilt Cell Imaging Shared Resource, supported by NIH grants OD021630, CA68485, DK20593, DK58404, DK59637, and EY08126.
- Islet and Pancreas Analysis Core and the Hormone Assay and Analysis Core of Vanderbilt Diabetes Research and Training Center (P30 DK020593).

## Supplementary Material

Supplemental data

Unedited blot and gel images

Supporting data values

## Version 1 06/15/2026

Electronic publication
