# Supplementary material for: Pancreatic islet α cell function and proliferation require the arginine transporter SLC7A2
Source: J Clin Invest. 2026 Jun 15;136(12):e173913. doi: 10.1172/JCI173913 (PMC13262736; doi:10.1172/JCI173913)

Working title: **Pancreatic islet α cell function and proliferation requires the arginine transporter SLC7A2**

Supplemental Figure Legends

Supplemental Figure 1. Physiological characterization of *Slc7a2*$^{-/-}$ mice. (A-C) IP glucose tolerance tests in **(A)** male, **(B)** female, and **(C)** male and female *Slc7a2*$^{+/+}$ (n=3 each gender), *Slc7a2*$^{+/-}$ (n=6 each gender) and *Slc7a2*$^{-/-}$ (n=3 each gender) mice. **(D-F)** Oral mixed meal tolerance tests in **(D)** male, **(E)** female, and **(F)** male and female *Slc7a2*$^{+/+}$ (n=4-8) and *Slc7a2*$^{-/-}$ (n=4-8) mice. **(G-I)** Blood glucose, serum glucagon and serum insulin for *Slc7a2*$^{+/+}$ (n=14), *Slc7a2*$^{+/-}$ (n=13) and *Slc7a2*$^{-/-}$ (n=12) mice after glucose stimulation. All antibody treatments for two weeks prior to sampling serum. Two way ANOVA was performed. Significance was designated: *$p<0.05$, **$p<0.005$, ***$p<0.0005$ and ****$p<0.0001$. **(J)** Analysis of serum amino acid concentrations in *Slc7a2*$^{+/+}$ (n=11), *Slc7a2*$^{+/-}$ (n=8) and *Slc7a2*$^{-/-}$ (n=12) mice. Asterisks indicate the 13 amino acids that were significantly higher in *Slc7a2*$^{-/-}$ animals than in *Slc7a2*$^{+/+}$ animals One way ANOVA was performed. Significance was p-value < 0.05.

Supplemental Figure 2. Gene expression characterization of *Slc7a2*$^{-/-}$ mouse islets. (A) Plot of individual RNAseq reads mapping to the *Slc7a2* locus from *Slc7a2*$^{+/+}$, *Slc7a2*$^{+/-}$ and *Slc7a2*$^{-/-}$ islets. Stable mRNA product lacking Exon 2 sequence but containing sequence from Intron 2 was detectable in *Slc7a2*$^{-/-}$ islets. **(B)** Quantitative real-time RT-PCR analysis of *Slc7a2* Exon 2 in RNA isolated from *Slc7a2*$^{-/-}$ mice and *Slc7a2*$^{+/+}$ controls (n=4 each). **(C-E)** Comparison of islet-specific cationic amino acid transporter expression **(C)**, islet transcription factor expression **(D)** and islet hormone expression **(E)** in *Slc7a2*$^{+/+}$ and *Slc7a2*$^{-/-}$ mice by bulk RNAseq of isolated islets. **(F)** Principal component analysis of bulk RNAseq data from *Slc7a2*$^{+/+}$ and *Slc7a2*$^{-/-}$ mice showing clustering by gender, but not by genotype.

Supplemental Figure 3. GCGR mAb-stimulated α cell proliferation does not alter stimulated glucagon or insulin secretion. (**A-C**) Blood glucose, serum glucagon and serum insulin for IgG treated *Slc7a2*$^{+/+}$ (n=4), *Slc7a2*$^{+/-}$ (n=4) and *Slc7a2*$^{-/-}$ (n=3) mice fasted for 6 hours, injected with glucose/arginine bolus and sampled 15 minutes post injection. (**D-F**) Blood glucose, serum glucagon and serum insulin for GCGR mAb treated *Slc7a2*$^{+/+}$ (n=4), *Slc7a2*$^{+/-}$ (n=5) and *Slc7a2*$^{-/-}$ (n=3) mice after glucose/arginine stimulation. All antibody treatments occurred for two weeks prior to sampling serum. Two way ANOVA was performed. Significance was designated: *p<0.05, **p<0.005, ***p<0.0005 and ****p<0.0001.

Supplemental Figure 4. Arginine stimulation of α cell proliferation is mTOR-dependent. (**A-D**) Immunostaining of GCGR mAb and IgG treated *Slc7a2*$^{+/+}$ and *Slc7a2*$^{-/-}$ mouse islets for glucagon and phospho-ribosomal protein S6 (P-S6$^{240/244}$), indicating active mTOR signaling (scale bars = 50μm; inset (**A'-D'**) scale bars = 10μm).

Supplemental Methods

Intraperitoneal Glucose Tolerance Test

To assess the role of SLC7A2 in glucagon and glucose tolerance, *Slc7a2*$^{+/+}$, *Slc7a2*$^{+/-}$ and *Slc7a2*$^{-/-}$ mice were fasted for 6 hours and then given intraperitoneal bolus of glucose to final concentrations of 2g / kg body weight. Blood was glucose was measured with a hand-held glucometer (Accu-Check Aviva) before injection (time 0) and at 15, 30, 60 and 120 minutes after injection.

Oral Mixed Meal Tolerance Test

To investigate how other nutrients and gluconeogenic substrates regulate glucose tolerance in *Slc7a2*$^{+/+}$, *Slc7a2*$^{+/-}$ and *Slc7a2*$^{-/-}$ mice were fasted for 6 hours and then given oral gavage of a bolus of original vanilla ensure (Abbott SKU#: 57243) (10μL/80% of b.w. in grams).

Blood was glucose was measured with a hand-held glucometer (Accu-Check Aviva) before injection (time 0) and at 15, 30, 60 and 120 minutes after injection.

Serum amino acid analysis

Serum amino acids were analyzed in the Vanderbilt Hormone and Analytical Services core by HPLC. Serum samples were prepared by deproteinization with sulfosalicylic acid and adding lithium loading buffer to adjust the pH to an ideal level. Amino acids were separated using lithium-based ion exchange with ninhydrin post-column and detected on a Biochrom 30 amino acid analyzer. Peak results were analyzed to procure quantitative results.

Whole Islet RNA sequencing

Whole Islets were pelleted and combined with to 20 µL lysis/binding solution in the RNAqueous micro-scale phenol-free total RNA isolation kit (Ambion). Trace DNA was removed with TURBO DNA-free (Ambion). RNA integrity was evaluated by Agilent 2100 Bioanalyzer. High integrity (RIN>7) total RNA was amplified, and cDNA libraries were constructed using the NEBNext® Poly(A) selection kit (New England Biolabs, Inc.). An Illumina NovaSeq 6000 was used to produce paired-end, 150-bp reads for each RNA sample yielding ~1.7billion total raw reads. 8 replicates were included for groups *Slc7a2*$^{+/-}$ and *Slc7a2*$^{-/-}$ whereas 7 replicates for *Slc7a2*$^{+/+}$. Paired end raw reads were aligned to the reference mouse genome mm10 (GRCm38) using The Spliced Transcripts Alignment to a Reference (STAR) version 2.6 (1). ~90% raw reads were uniquely mapped to genomic sites. Next, alignment quality and transcript quantification were performed using Strand NGS analysis platform 3.4 (Strand Life Sciences, India). Raw counts was normalized for library size and differential expression analysis using DESeq2 1.26 (2) with sex as covariate. Very lowly expressed genes were discarded from the analysis, keeping only genes covered by at least 20 reads in a minimum of three samples. Differentially expressed genes were defined by P_{adj} <0.05. RNA Sequencing data have been deposited in NCBI GEO. RNAseq analysis confirmed stable *Slc7a2* mRNA in *Slc7a2*$^{-/-}$ islets. Analysis of individual reads from RNAseq showed no reads in exon 2, the original knockout

target, but some reads in intron 2 (Supplemental Fig 2A). Despite the presence of some RNA from this locus, the phenotypic results from these and previous studies indicate homozygous mutant mice are functionally null at the *Slc7a2* locus.

Supplemental References

1. Dobin A, Davis CA, Schlesinger F, Drenkow J, Zaleski C, Jha S, et al. STAR: ultrafast universal RNA-seq aligner. *Bioinformatics.* 2013;29(1):15-21.
2. Love MI, Huber W, and Anders S. Moderated estimation of fold change and dispersion for RNA-seq data with DESeq2. *Genome Biol.* 2014;15(12):550.

Supplemental Table 1: Amino acid concentrations in culture media for *ex vivo* islet α cell proliferation experiments.

	RPMI 1640-based Media, Amino Acid Concentrations (μM)									
**Amino Acids**	**All AA -**	**All AA +**	**AIKMVOPSTYRHGN -**	**AIKMV -**	**OPSTY -**	**RHGN -**	**RHGN - / R +**	**RHGN - / H +**	**RHGN - / G +**	**RHGN - / N +**
Glycine	200	**1500**	200	**1500**	**1500**	200	200	200	**1500**	200
L-Arginine	40	**600**	40	**600**	**600**	40	**600**	40	40	40
L-Asparagine	40	**350**	40	**350**	**350**	40	40	40	40	**350**
L-Aspartic acid	10	**10**	**10**	**10**	**10**	**10**	**10**	**10**	**10**	**10**
L-Cystine 2HCl	10	**10**	**10**	**10**	**10**	**10**	**10**	**10**	**10**	**10**
L-Glutamic Acid	100	**300**	**300**	**300**	**300**	**300**	**300**	**300**	**300**	**300**
L-Glutamine	500	**3250**	**3250**	**3250**	**3250**	**3250**	**3250**	**3250**	**3250**	**3250**
L-Histidine	40	**250**	40	**250**	**250**	40	40	**250**	40	40
L-Isoleucine	125	**250**	125	125	**250**	**250**	**250**	**250**	**250**	**250**
L-Leucine	225	**400**	**400**	**400**	**400**	**400**	**400**	**400**	**400**	**400**
L-Lysine hydrochloride	200	**1200**	200	**1200**	**1200**	**1200**	**1200**	**1200**	**1200**	**1200**
L-Methionine	60	**280**	60	60	**280**	**280**	**280**	**280**	**280**	**280**
L-Phenylalanine	75	**75**	**75**	**75**	**75**	**75**	**75**	**75**	**75**	**75**
L-Proline	85	**400**	85	**400**	85	**400**	**400**	**400**	**400**	**400**
L-Serine	100	**1250**	100	**1250**	100	**1250**	**1250**	**1250**	**1250**	**1250**
L-Threonine	150	**1750**	150	**1750**	150	**1750**	**1750**	**1750**	**1750**	**1750**
L-Tryptophan	65	**65**	**65**	**65**	65	**65**	**65**	**65**	**65**	**65**
L-Tyrosine	50	**250**	50	**250**	50	**250**	**250**	**250**	**250**	**250**
L-Valine	300	**600**	300	300	**600**	**600**	**600**	**600**	**600**	**600**
L-Alanine	350	**2250**	350	350	**2250**	**2250**	**2250**	**2250**	**2250**	**2250**
L-Ornithine	100	**400**	100	**400**	100	**400**	**400**	**400**	**400**	**400**
Data presented	Figures 1A, 4B and Supp. Figure 1A		Supp. Fig 1A			Figure 1A and Supp. Fig 1A	Figure 1A			

Bold numbers indicate high amino acid concentrations.

Supplemental Table 2: Amino acid concentrations in αTC1-6 culture media for cell growth assays.

	Media, Amino Acid Concentrations (μM)				
**Amino Acids**	**α TC1-6 Culture**	**No Gln / No Arg**	**No Gln / 0.4mM Arg**	**4mM Gln / No Arg**	**4mM Gln / 0.4mM Arg**
Glycine	400.0	400.0	400.0	400.0	400.0
L-Arginine	398.1	0.0	**398.1**	0.0	**398.1**
L-Cystine 2HCl	201.3	201.3	201.3	201.3	201.3
L-Glutamine	4000.0	0.0	0.0	**4000.0**	**4000.0**
L-Histidine	200.0	200.0	200.0	200.0	200.0
L-Isoleucine	801.5	801.5	801.5	801.5	801.5
L-Leucine	801.5	801.5	801.5	801.5	801.5
L-Lysine hydrochloride	797.8	**797.8**	**797.8**	**797.8**	**797.8**
L-Methionine	201.3	201.3	201.3	201.3	201.3
L-Phenylalanine	400.0	400.0	400.0	400.0	400.0
L-Serine	400.0	400.0	400.0	400.0	400.0
L-Threonine	798.3	798.3	798.3	798.3	798.3
L-Tryptophan	78.4	78.4	78.4	78.4	78.4
L-Tyrosine	398.5	398.5	398.5	398.5	398.5
L-Valine	803.4	803.4	803.4	803.4	803.4
Base Medium	DMEM, Low Glucose (Gibco, 11885-084)	SILAC DMEM Flex Medium (Gibco Cat. No. A2493901) *Supplemented with glucose, pyruvate, HEPES and BSA to match α TC1-6 Medium, See Methods			
Application	General α TC1-6 Culture and shRNA growth assay	αTC1-6 arginine/glutamine growth assay			
Data presented	Figure 4A	Figure 1B			

Bold numbers amino acid supplemented into base medium.

Supplemental Table 3: Top 50 solute carriers in Human, Mouse and Zebrafish α and β cells from highest to lowest relative expression.

Human

Gene Symbol	Average TMM Normalized Expression (x 10^{-2}) in α cells	Gene Symbol	Average TMM Normalized Expression (x 10^{-2}) in β cells
SLC7A2 *	458.61	*SLC30A8*	194.25
SLC30A8	188.34	*SLC7A2* *	134.41
SLC7A8	86.79	*SLC7A8*	78.07
SLC7A14	43.56	*SLC6A6*	67.67
SLC38A4	40.13	*SLC2A13*	66.82
SLC23A2	25.25	*SLC18A2*	40.89
SLC35F5	23.86	*SLC6A17*	31.68
SLC25A3	23.51	*SLC39A14*	27.86
SLC7A1	23.36	*SLC7A1*	27.59
SLC2A13	21.56	*SLC39A9*	25.88
SLC25A5	21.49	*SLC4A10*	25.71
SLC36A4	20.98	*SLC25A3*	24.42
SLC11A2	20.77	*SLC23A2*	23.98
SLC4A10	19.20	*SLC25A36*	22.40
SLC29A1	17.63	*SLC11A2*	22.32
SLC22A17	17.45	*SLC29A1*	20.03
SLC38A2	17.33	*SLC38A2*	17.84
SLC25A4	17.25	*SLC31A1*	17.29
SLC9A7	16.96	*SLC24A2*	17.24
SLC41A1	15.96	*SLC9A7*	16.80
SLC31A1	15.75	*SLC1A4*	15.23
SLC18A2	15.43	*SLC8A1*	15.12
SLC25A44	15.16	*SLC39A6*	14.96
SLC6A17	14.92	*SLC35F5*	13.74
SLC25A36	14.62	*SLC20A2*	13.50
SLC39A14	14.36	*SLC12A6*	13.27
SLC17A5	13.90	*SLC25A44*	13.20
SLC39A6	13.75	*SLC17A5*	12.97
SLC29A4	13.74	*SLC41A1*	12.48
SLC39A10	13.10	*SLC22A17*	12.39
SLC6A4	13.06	*SLC25A4*	12.14
SLC25A53	12.82	*SLC7A14*	12.04
SLC30A9	12.65	*SLC30A9*	11.41
SLC4A8	12.55	*SLC4A8*	11.08
SLC9A3R1	12.48	*SLC36A4*	10.95
SLC5A1	11.68	*SLC30A7*	10.79
SLC9A8	11.17	*SLC9A3R1*	10.24
SLC41A2	11.07	*SLC38A4*	10.23
SLC24A1	10.46	*SLC39A10*	10.19
SLC3A2	9.78	*SLC9A8*	10.00
SLC50A1	9.72	*SLC4A4*	9.93
SLC25A51	9.45	*SLC46A3*	9.80
SLC38A10	9.30	*SLC35E1*	9.65
SLC30A7	8.88	*SLC4A1AP*	9.56
SLC20A2	8.49	*SLC9A3R1*	9.49
SLC4A1AP	8.47	*SLC9A8*	9.02
SLC1A4	8.35	*SLC33A1*	8.95
SLC16A12	8.34	*SLC25A5*	8.93
SLC6A6	8.11	*SLC3A2*	8.84
SLC16A7	7.82	*SLC7A5*	8.82

Brissova, *et al.*, *Cell Reports*, 2018
Saunders, *et al., Cell Metabolism*, 2018

Mouse

Gene Symbol	Average RPKM Normalized Expression in α cells	Gene Symbol	Average RPKM Normalized Expression in β cells
Slc30a8	398.15	*Slc25a3*	244.28
Slc25a4	200.10	*Slc25a4*	205.28
Slc38a4	185.96	*Slc25a39*	189.33
Slc7a8	177.28	*Slc30a8*	183.33
Slc25a3	174.51	*Slc3a2*	155.52
Slc7a2 *	160.16	*Slc39a7*	150.43
Slc3a2	122.14	*Slc35b1*	120.99
Slc25a5	120.06	*Slc7a5*	119.79
Slc25a39	118.23	*Slc25a5*	100.57
Slc39a7	84.62	*Slc25a1*	100.03
Slc35b1	67.95	*Slc38a4*	94.51
Slc16a10	63.00	*Slc2a5*	80.89
Slc25a1	56.09	*Slc7a14*	70.78
Slc38a10	51.16	*Slc38a10*	69.86
Slc40a1	49.16	*Slc50a1*	69.06
Slc50a1	41.45	*Slc7a8*	60.54
Slc16a12	41.24	*Slc35f5*	58.86
Slc35g2	39.94	*Slc33a1*	50.95
Slc7a14	39.00	*Slc6a6*	46.64
Slc38a5	38.27	*Slc37a4*	45.76
Slc25a17	36.32	*Slc25a11*	42.82
Slc6a6	35.36	*Slc25a44*	41.55
Slc48a1	33.72	*Slc35g2*	40.55
Slc43a2	33.30	*Slc48a1*	39.38
Slc25a11	33.23	*Slc2a1*	37.90
Slc37a1	33.12	*Slc25a12*	35.82
Slc2a5	29.02	*Slc25a17*	35.70
Slc30a9	28.75	*Slc4a7*	35.04
Slc39a11	27.42	*Slc25a51*	34.98
Slc33a1	27.10	*Slc30a9*	34.35
Slc6a17	27.04	*Slc30a5*	33.92
Slc30a5	25.21	*Slc43a2*	32.50
Slc25a51	25.16	*Slc16a12*	32.29
Slc25a12	24.96	*Slc38a2*	31.98
Slc35f5	24.60	*Slc16a10*	29.84
Slc38a2	22.95	*Slc29a1*	28.91
Slc29a1	22.67	*Slc35b4*	28.72
Slc41a2	22.59	*Slc7a2* *	26.60
Slc7a5	22.55	*Slc6a17*	26.12
Slc25a44	21.90	*Slc25a23*	20.66
Slc4a7	21.57	*Slc40a1*	20.53
Slc35b4	21.17	*Slc37a1*	20.17
Slc29a4	20.83	*Slc35a1*	18.87
Slc37a4	20.78	*Slc4a2*	17.99
Slc31a2	20.67	*Slc31a2*	14.79
Slc22a17	20.36	*Slc22a17*	12.74
Slc25a23	20.08	*Slc39a11*	12.13
Slc4a2	19.92	*Slc29a4*	11.73
Slc2a1	19.46	*Slc41a2*	3.86
Slc35a1	18.97	*Slc38a5*	1.33

DiGruccio MR, *et al.*, Molecular Metabolism, 2016

Zebrafish

Gene Symbol	Average TMM Normalized Expression (x 10^{-2}) in α cells	Gene Symbol	Average TMM Normalized Expression (x 10^{-2}) in β cells
slc1a4	598.10	*slc5a10*	874.19
slc43a2b	321.03	*slc1a4*	418.50
slc7a2 *	234.35	*slc43a2b*	308.55
slc5a10	560.77	*slc7a2* *	204.68
slc3a2a	191.39	*slc2a2*	179.25
slc25a14	94.33	*slc25a3b*	167.24
slc3a2b	98.19	*slc3a2a*	165.58
slc9a3r1	121.98	*slc30a2*	143.16
slc16a9b	70.22	*slc9a3r1*	128.21
slc2a2	142.86	*slc39a7*	127.71
slc25a3b	136.36	*slc7a8b*	84.31
slc20a1b	66.57	*slc3a2b*	78.16
slc30a2	105.37	*slc31a1*	71.27
slco5a1	44.52	*slc25a14*	61.74
slc35g2a	55.73	*slc30a7*	54.02
slc6a6a	49.19	*slc35g2a*	51.12
slc43a2a	48.56	*slc25a36b*	47.99
slc39a7	87.87	*slc43a2a*	44.89
slc25a33	34.39	*slc20a1b*	43.66
slc35g1	36.08	*slc17a9b*	42.42
slc16a6b	23.27	*slc17a7 (3)*	42.10
slc17a7 (3)	41.01	*slc39a6*	41.80
slc18a2	32.28	*slc6a6a*	38.57
slc7a8b	60.63	*slc25a20*	36.73
slc30a7	44.09	*slc16a9b*	32.85
slc25a22	26.12	*slc37a4a*	31.30
slco5a1 (2)	29.95	*SLC35G1*	30.91
slc26a5	19.83	*slc25a36a*	30.54
slc31a1	51.02	*slco5a1 (2)*	27.71
slc25a6	24.79	*slc9a8*	26.97
slc25a36a	28.47	*SLC25A24*	26.35
slc30a9	25.54	*slc25a33*	26.00
slc20a2	21.93	*slc18a2*	25.55
slc25a25b	23.52	*slc30a9*	25.27
slc38a10	20.15	*slco5a1*	25.25
slc17a9b	33.52	*slc30a5*	24.98
slc12a9	21.56	*slc38a2*	24.83
slc25a36b	35.25	*slc39a10*	22.82
slc25a32a	16.29	*slc25a25b*	22.05
slc25a38b	20.27	*slc25a12*	21.98
slc4a2b	17.80	*slc6a17*	21.79
slc25a20	29.31	*slc25a6*	20.09
slc16a10	18.82	*slc25a22*	19.49
slc9a6a	17.27	*slc7a10a*	19.13
slc12a7b	14.87	*slc20a2*	18.62
slc9a7	16.83	*slc12a9*	18.56
slc37a4a	25.54	*slc36a1*	18.45
slc29a4	18.60	*slc25a38b*	18.20
slc25a12	20.82	*slc29a4*	17.48
slc17a5	16.46	*slc7a4*	16.99

Tarifeño-Saldivia, *BMC Biology*, 2017

Supplemental Figure 1

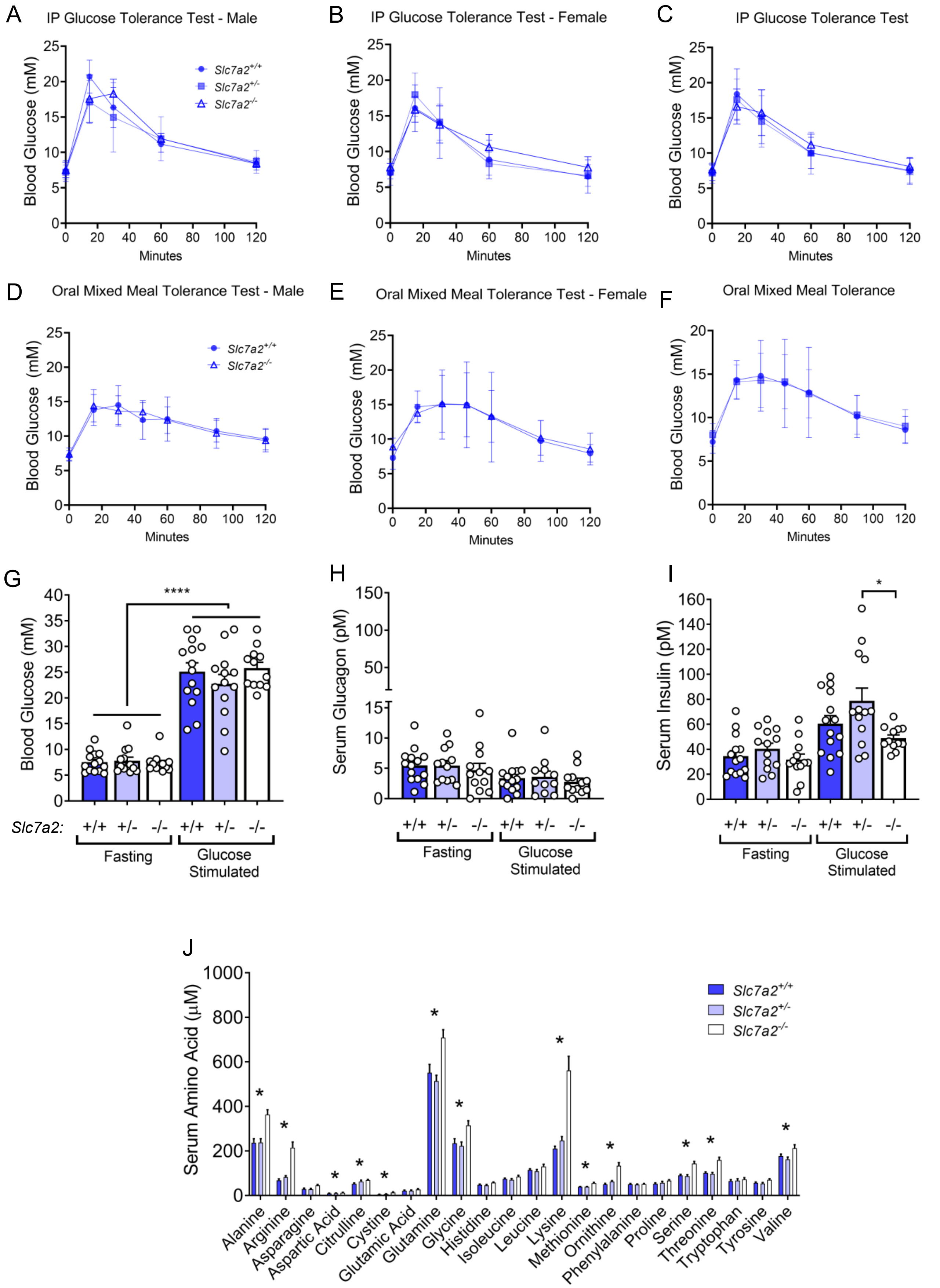

Supplemental Figure 2

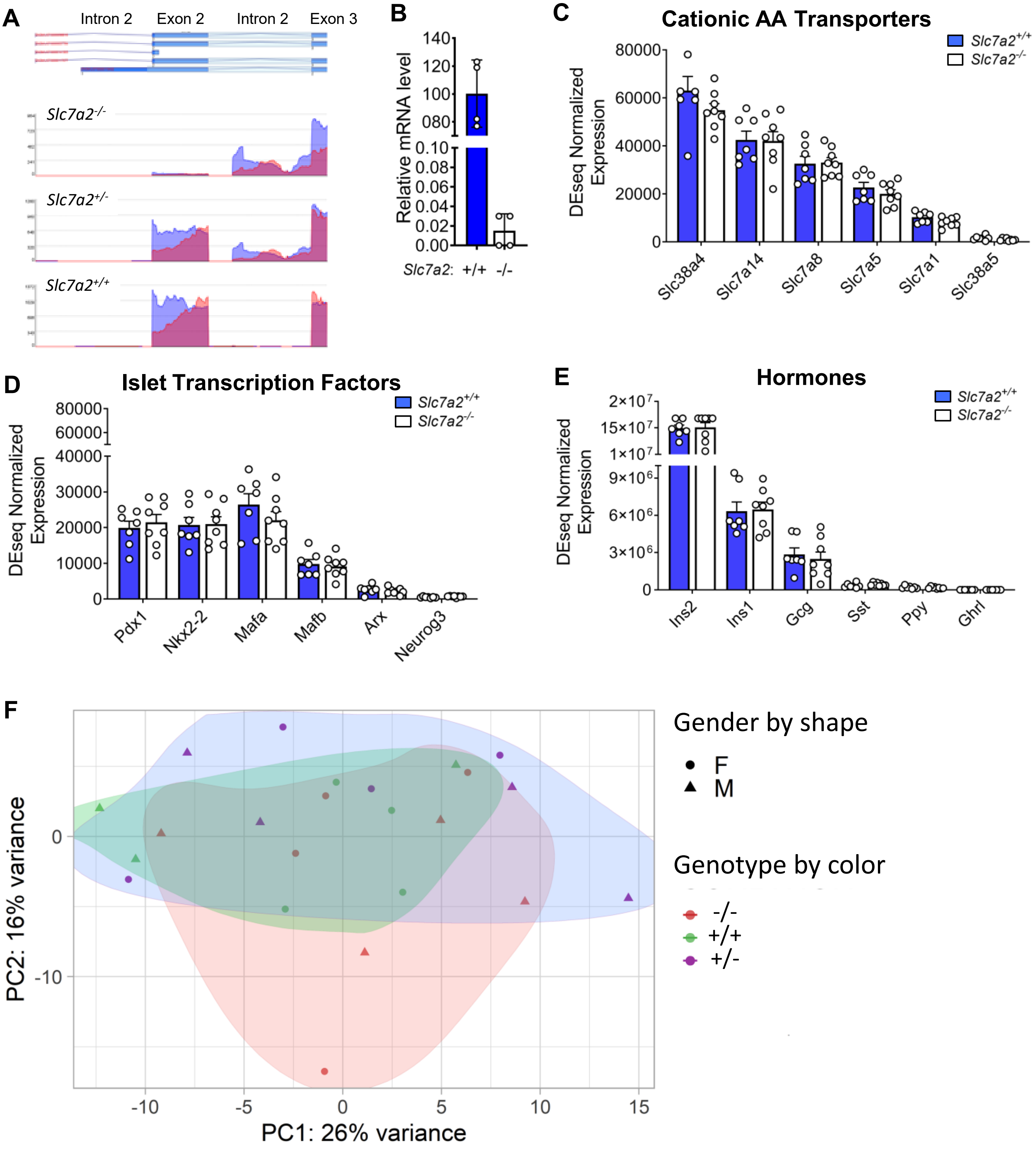

Supplemental Figure 3

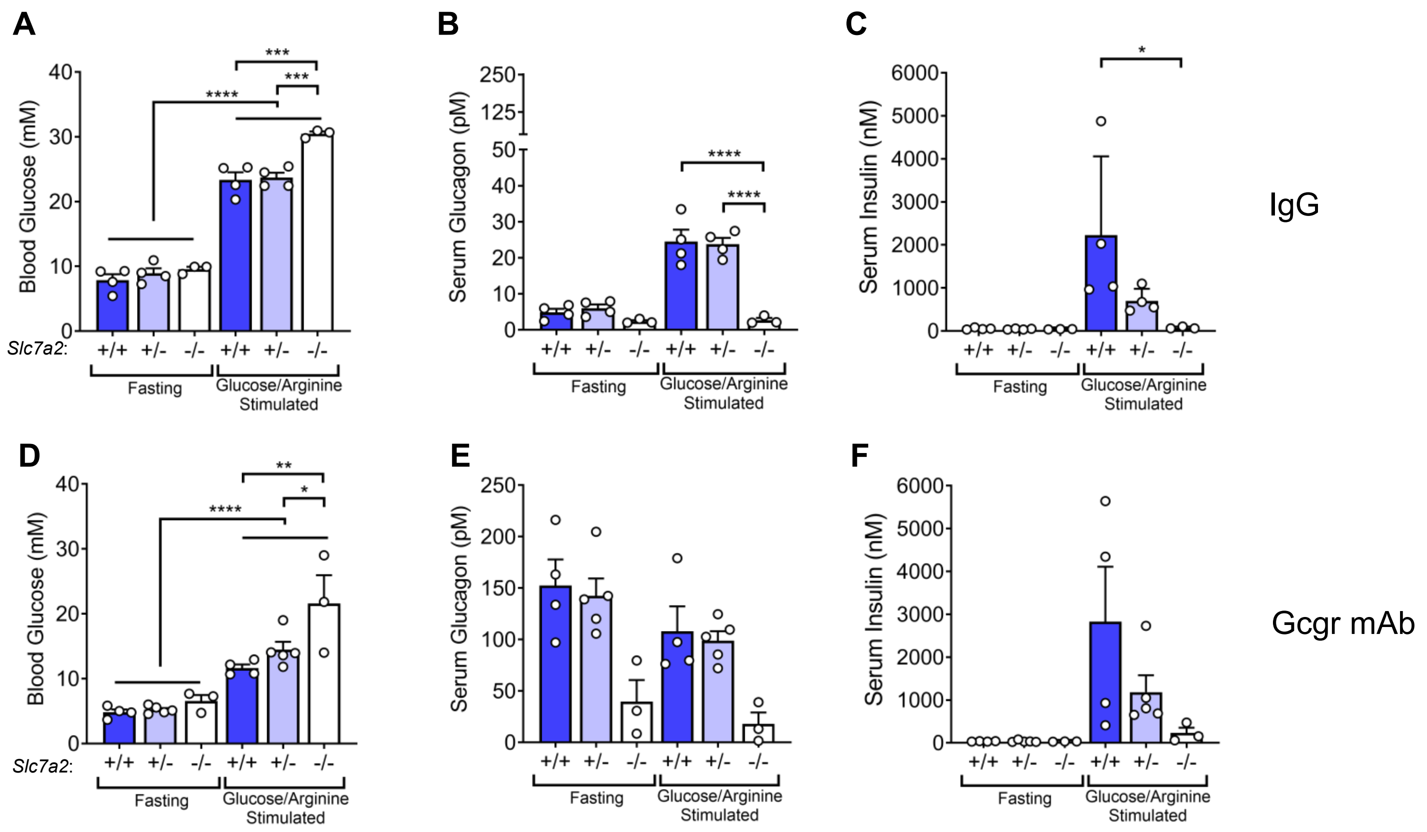

Supplemental Figure 4

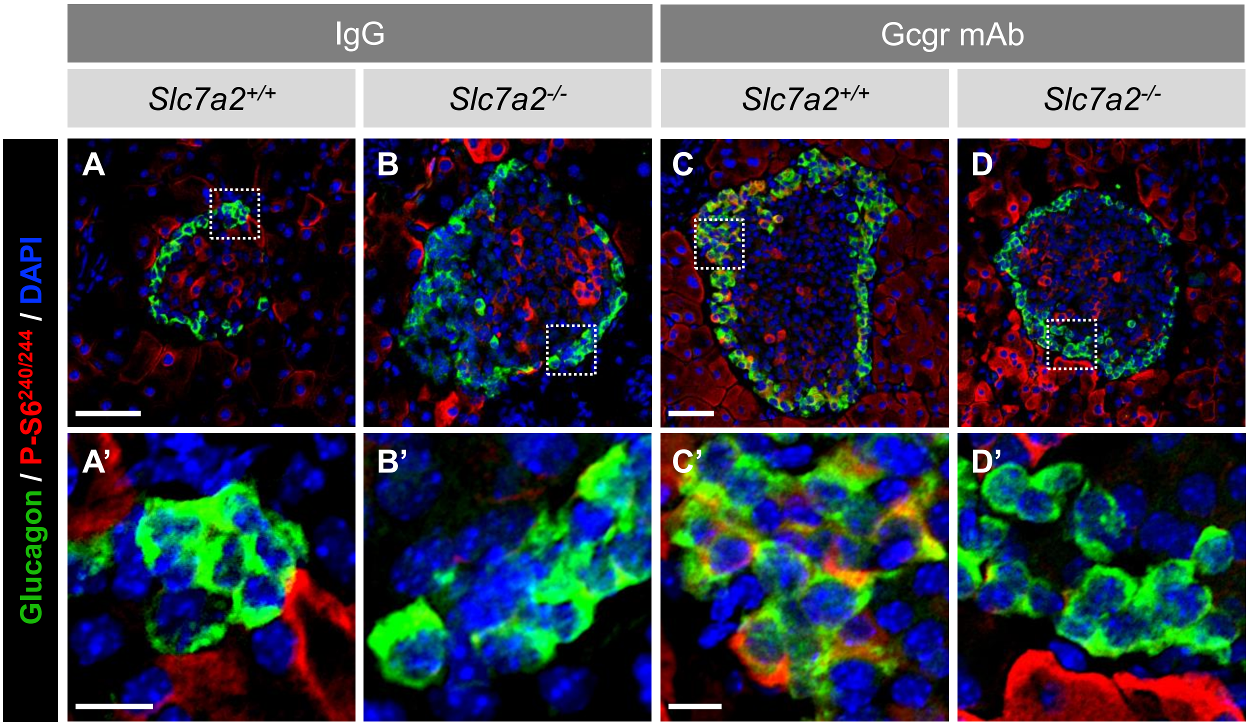

Supplement: Supplemental data [file jci-136-173913-s019.pdf]
