# Supplementary material for: Pancreatic islet α cell function and proliferation require the arginine transporter SLC7A2
Source: J Clin Invest. 2026 Jun 15;136(12):e173913. doi: 10.1172/JCI173913 (PMC13262736; doi:10.1172/JCI173913)
Supplement: Unedited blot and gel images [file jci-136-173913-s020.pdf]

Untouched blots in Figure 5 *Spears et al*

Blot #1

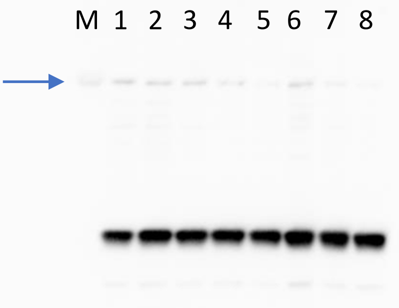

Non-specific band from GAPDH probing

GAPDH band in figure

Blot #2

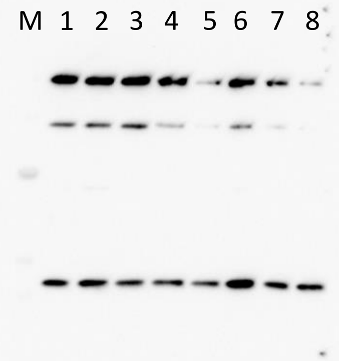

SLC7A2 band (in figure and for densitometry)

Non-specific band from GAPDH probing

Marker band (~50kDa)

GAPDH band

* Probed for GAPDH first (Blot #1), then for SLC7A2 (Blot #2), thus residual GAPDH and non-specific band on SLC7A2 blot

Lanes 1-5 contain aTC1-6 cell lysates from other rounds of clonal selection that were less efficient at knocking down SLC7A2 expression and their untreated or scrambled controls to be used as a comparison to this round. These are not related to the studies presented in this manuscript.

Lanes 6-8 contain the samples used to verify knockdown in the clones used for experiments presented here.

6 Scramble shRNA

7 shRNA *Slc7a2* C1

8 shRNA *Slc7a2* C2
